# Tetra-substituted BDPA radicals *via* click-chemistry and application to liquid-state DNP

**DOI:** 10.1039/d6cc01089j

**Published:** 2026-05-13

**Authors:** Iram M. Ahmad, Pralambika Roy, Andrei Kuzhelev, Snorri Th. Sigurdsson

**Affiliations:** a University of Iceland, Department of Chemistry, Science Institute, Dunhaga 3 Reykjavik 107 Iceland snorrisi@hi.is; b Institute of Physical and Theoretical Chemistry and Center for Biomolecular Magnetic Resonance (BMRZ), Goethe University 60438 Frankfurt am Main Germany

## Abstract

The 1,3-bisdiphenylene-2-phenylallyl (BDPA) radical is a promising polarizing agent for DNP NMR, but is limited by poor persistence. A divergent synthetic strategy, using copper(i)-catalyzed azide–alkyne cycloaddition, is presented for preparing tailored BDPA derivatives. A high-molecular-weight, sterically shielded BDPA-dendrimer showed improved persistence and the highest liquid-state DNP enhancement reported thus far.

## Abstract 2

A divergent CuAAC strategy for the synthesis of BDPA radicals with tunable size and properties is reported. A high-molecular-weight BDPA dendrimer exhibited the highest solution-state DNP ^1^H-NMR enhancement in viscous liquid reported to date.

## Graphical abstract

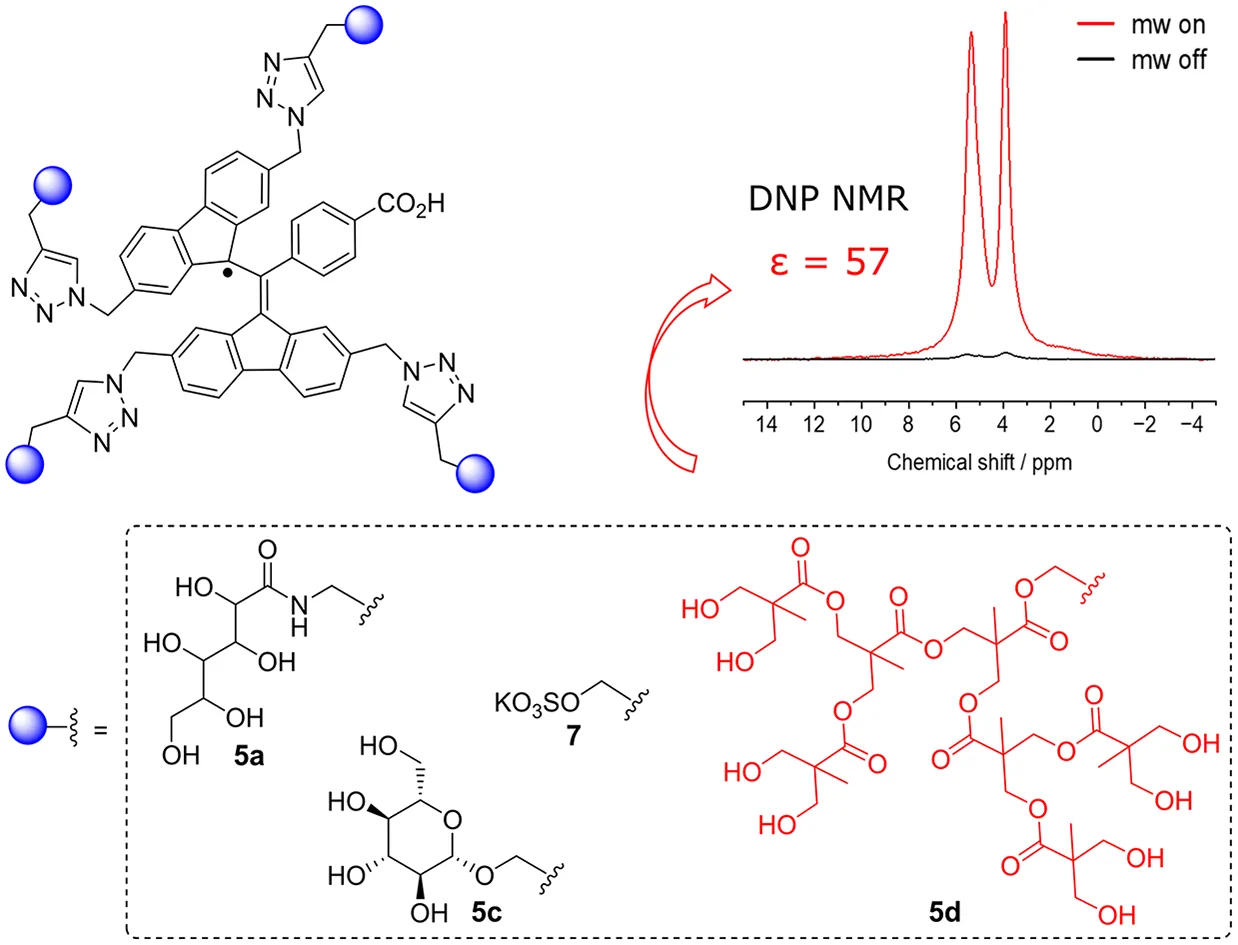

Nuclear magnetic resonance (NMR) spectroscopy is an important analytical technique for investigating the structure and dynamics of biomolecules and materials.^1–4^ However, a major drawback of NMR is its low sensitivity, which is due to the small energy difference between the ground and excited states of nuclear spins in a magnetic field. Dynamic nuclear polarization (DNP) NMR has emerged as a powerful technique to overcome this drawback, by transferring the much higher polarization of unpaired electrons to the nuclei of interest.^5–7^ In DNP NMR, the sample is doped with a paramagnetic molecule, referred to as a polarizing agent, and irradiated with microwaves (µw) during signal acquisition.^8,9^ Persistent organic radicals are the most commonly used polarizing agents.

Bis-nitroxide biradicals are a class of polarizing agents that are fairly easy to synthesize and have extensively been used for DNP at magnetic fields of 9.4 and 14.1 T.^10–14^ AsymPol-POK (Fig. 1) is a prominent example for solid-state DNP in aqueous solutions, offering high DNP performance at these magnetic fields.^12,15^ With recent technological advances, DNP NMR has been extended to even higher magnetic fields (≥18.8 T) in order to improve both the signal-to-noise ratio and signal resolution.^16^ However, the performance of nitroxides drops at very high magnetic fields due to shorter electronic relaxation times and a concomitant broader EPR signal, which broadens linearly with the external magnetic field.^16,17^ This results in reduced excitation of the electronic spins and thus, lower DNP enhancement. Moreover, loss of nuclear polarization through depolarization becomes prominent for nitroxides at very high fields.^18^

**Fig. 1:**
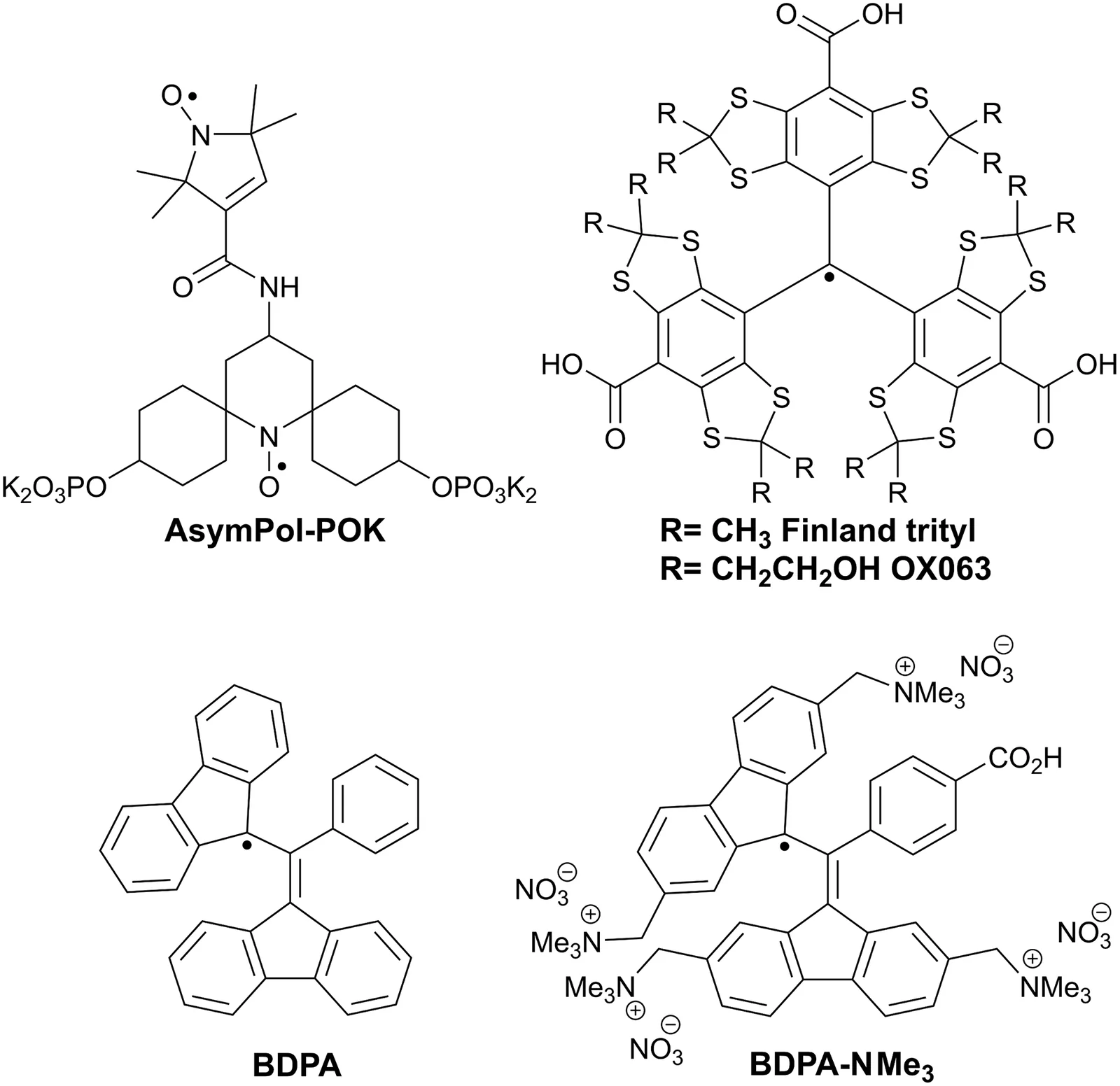
Persistent radicals used as polarizing agents for DNP NMR: AsymPol-POK, Finland trityl, OX063, BDPA and trimethylammonium BDPA (BDPA-NMe_3_).

To overcome the drawbacks associated with nitroxides, carbon-based radicals like Finland trityl^18–20^ and 1,3-bisdiphenylene-2-phenylallyl (BDPA)^21^ (Fig. 1) have attracted attention as promising radicals for high field DNP-NMR. The isotropic *g*-values of carbon lead to a much lower or even non-existing depolarization.^18,21^ Furthermore, the narrow EPR line of carbon radicals in the solid-state and their long electron spin–lattice relaxation times (*T*_1e_) enable efficient saturation at comparatively lower microwave power.^16,22^

An advantage of the BDPA radical over the Finland trityl is its relative ease of synthesis.^23,24^ Moreover, its EPR signal is narrower and more isotropic than the Finland trityl radical.^22^ BDPA radicals have been used for DNP in both liquids and solids,^22,25–31^ where they exhibit multiple polarization pathways. Traditionally, the Overhauser effect (OE) was considered exclusive to liquids, relying on fast molecular motion to mediate electron-nuclear cross-relaxation.^32,33^ However, BDPA was shown to have the ability to operate in solids through the OE.^26,27^ This behaviour has been linked to its mixed-valence character and intramolecular charge-transfer dynamics.^34^ Furthermore, Kuzhelev *et al*. have also shown that BDPA monoradicals can polarize fluid lipid membranes and analytes in viscous solutions through the solid effect (SE) mechanism at high magnetic fields, previously only observed in solids.^29,30^ When molecular tumbling of an analyte in solution is reduced,^30,35^ electron-nuclear dipolar interactions slow down, which allows efficient polarization transfer and sizable signal enhancements.^36^ Such polarization can also be observed for large molecules (with a long rotational correlation time) in aqueous solutions.^29,37^ Thus, BDPA radicals exhibit versatile and interesting DNP properties for solid- and solution-state alike. Nonetheless, BDPA radicals have limitations.

Two major drawbacks of BDPA-based radicals have been their low persistence and limited solubility in aqueous solutions for use in structural biology.^38^ A new class of tetraalkylammonium BDPA derivatives has partially addressed these shortcomings by providing more persistent radicals with tuneable solubility.^39^ However, the water-soluble trimethylammonium BDPA derivative (BDPA-NMe_3_) (Fig. 1), has limited persistence in aqueous solutions, probably due to the tendency of tetraalkylammonium salts to aggregate in water,^40–42^ leading to dimerization.^38,39^ Attaching bulky substituents to BDPA should reduce its tendency to aggregate and thereby enhance its stability.^43,44^ Here we describe a convergent synthetic strategy that enables conjugation of a variety of different substituents to BDPA by Cu-catalyzed azide–alkyne cycloaddition (CuAAC) with a focus on the incorporation of hydrophilic and sterically demanding groups. This approach provides flexibility in preparing various substituted BDPA derivatives with tailored properties and complements the strategy of increasing the persistence of BDPA by changing the electronic properties of the aromatic rings.^45,46^ Of the BDPA derivatives described here, a BDPA-conjugated dendrimer was particularly promising, with improved persistence in water and the highest reported DNP enhancement thus far in viscous liquids.

The synthetic strategy was based on the known tetrabromo BDPA derivative 1^39^ and its conversion to tetraazide BDPA 2 (Scheme 1). This tetraazide can be readily conjugated to various alkyne-bearing substituents by CuAAC, including hydrophilic and sterically demanding groups. We chose four different alkynes with distinct properties: propargyl alcohol (3a), which can be further derivatized; a polyhydroxyamide derivative (3b)^47^ to use in sorbitol-based glass matrices for DNP; a glucose derivative (3c)^48^ as a neutral and hydrophilic moiety that could provide some steric shielding; and a dendrimer alkyne (3d) to impart solubility and extensive steric effects (Scheme 1).

**Scheme 1:**
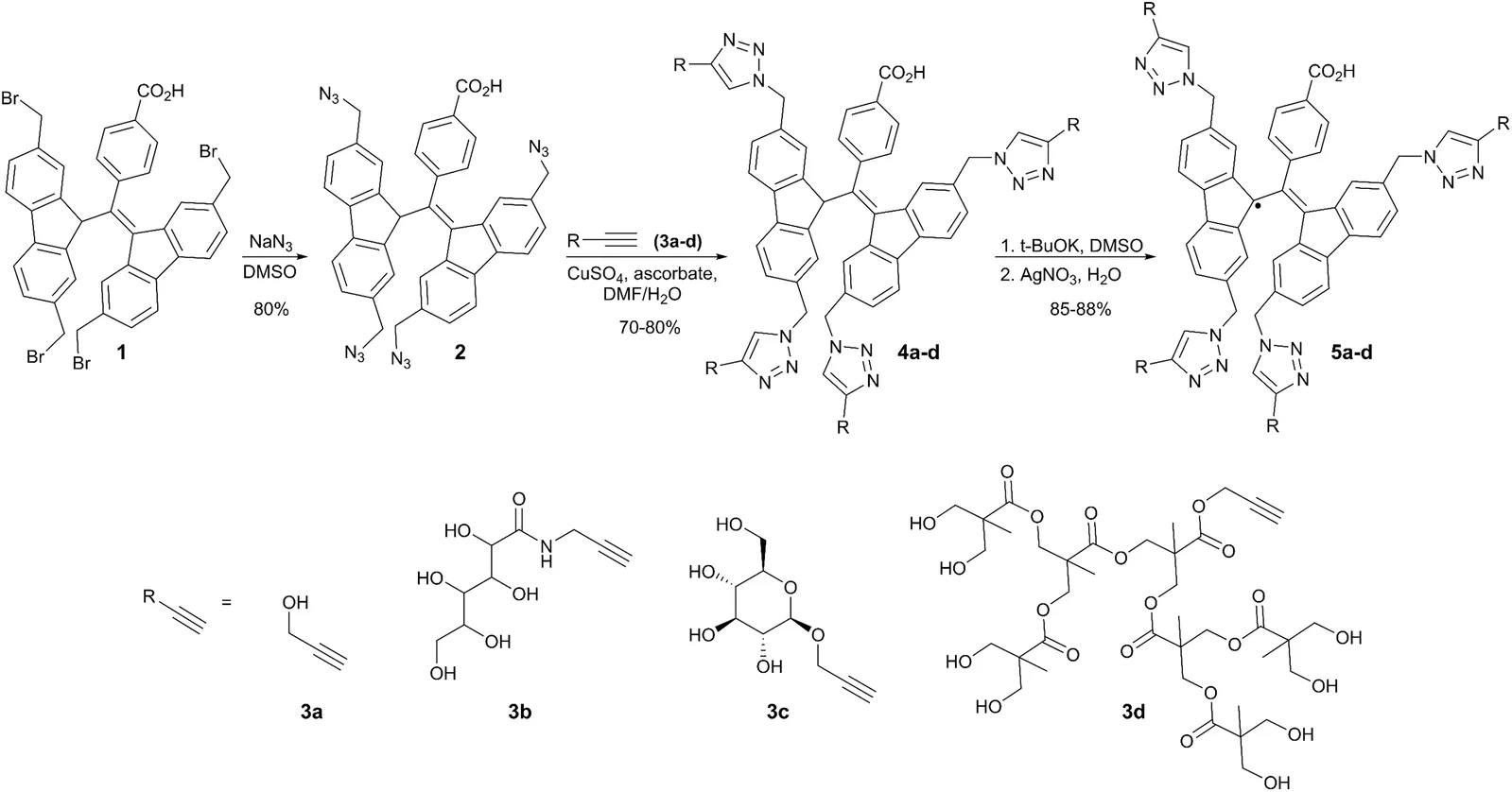
Synthesis of water-soluble BDPA radicals 5a–d*via* CuAAC of tetraazide 2 and their corresponding alkyne substrates 3a–d.

The synthesis began with the azidation of the tetrabromide 1^39^ to give the tetraazide BDPA derivative 2 in excellent yield (Scheme 1). The click reaction of 2 with the alkynes (3a–d) was straightforward, however, the purification of BDPA derivatives 4a–d was challenging due to either limited solubility (4a and 4b) or very high polarity (4c and 4d). For derivatives 4a, 4b and 4c, precipitation with Et_2_O gave fairly pure products with good yields. Compound 4d was purified by flash column chromatography. The corresponding BDPA radicals 5a–d were prepared by treating derivatives 4a–d sequentially with *t*-BuOK and AgNO_3_ (Scheme 1). Since compound 4d was prone to hydrolysis in the presence of base, a shorter reaction time was used for the deprotonation with *t*-BuOK (see SI for details).

BDPA radicals 5a–d were all soluble in DMSO, while only 5c and 5d were soluble in water. On the other hand, compound 5b was unexpectedly insoluble in water, despite being a sugar-based derivative like 5c. The insolubility of 5b in water is likely due to a combination of intermolecular hydrogen bonding involving the carbohydrate moieties and π–π stacking of the BDPA cores. As anticipated, the tetrahydroxyl BDPA radical 5a exhibited negligible solubility in water. However, the hydroxyl groups can be readily phosphorylated^12^ or converted to sulfates to dramatically increase hydrophilicity.^49^ Thus, the tetrasulfate derivative of 4a was prepared and subsequently converted to the corresponding radical 7 in good overall yield (Scheme 2). The sulfate derivative 7 showed excellent solubility in water.

**Scheme 2:**
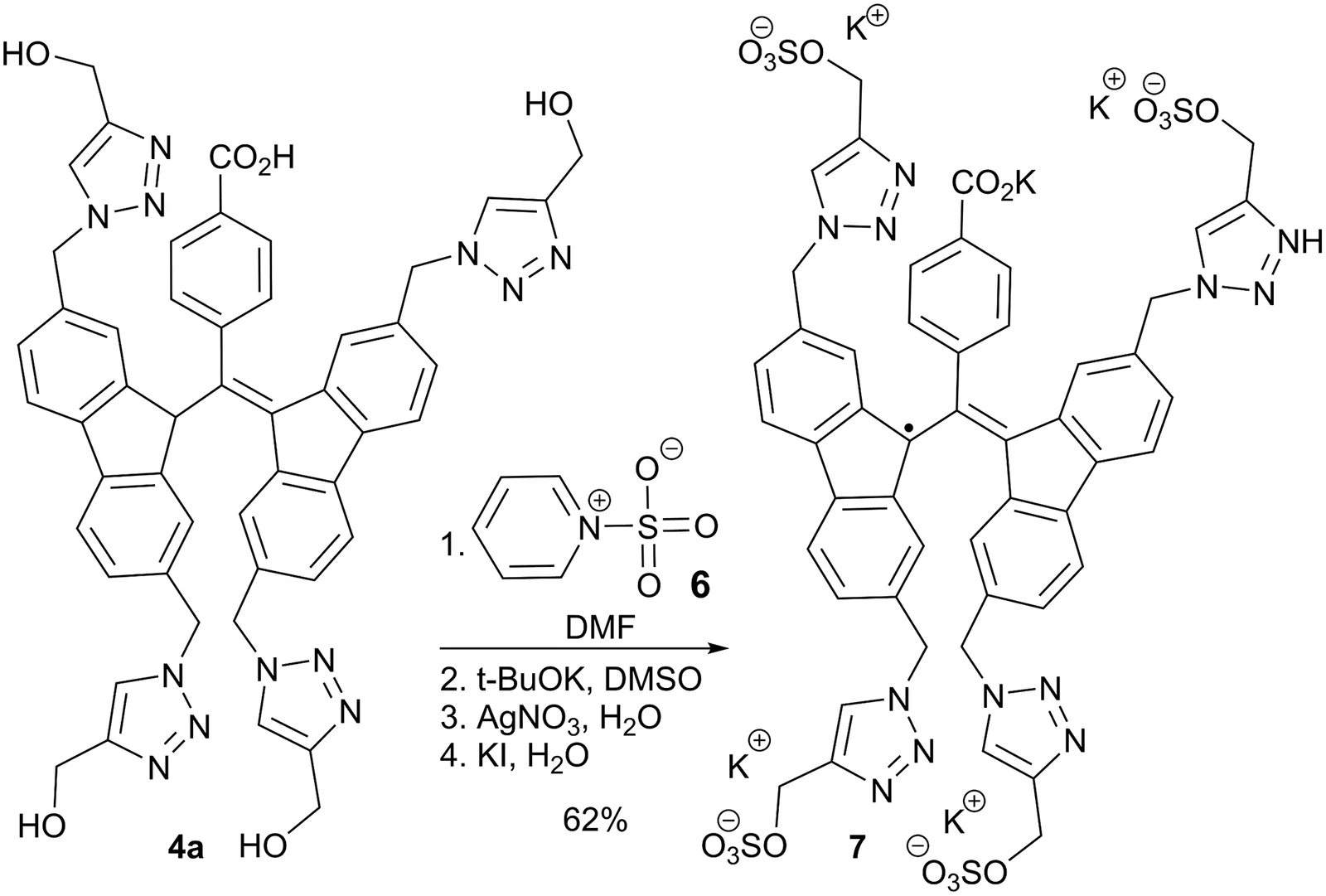
Synthesis of BDPA-sulfate 7 from 4a.

Based on its high molecular weight and good solubility in aqueous solutions, the BDPA-dendrimer radical (5d) was chosen for investigation of persistence and for evaluation as a polarizing agent. The persistence of 5d in DMSO and water was determined by monitoring the radical concentration as a function of time by UV-Vis spectroscopy (Fig. 2).^39^ Interestingly, the radical concentration 5d unexpectedly increased in DMSO during the first three days, before reaching a plateau (Fig. 2A). This indicated that the that the radical precursor 4d, which was still present in the sample of 5d, was converted to the radical under these conditions. DMSO plays a key role, facilitating formation of the BDPA anion (change in color), which then converts to the radical. The same behaviour was also observed when 4d was dissolved in DMSO, which ruled out possibility of oxidation of 5d by residual oxidizing agent from the previous step (Fig. S27). Once the anion had been converted to the radical, it remained persistent in DMSO for at least 25 days (Fig. 2A).^39^ The estimated half-life of 5d in water was ∼2 days, which was substantially longer that of BDPA-NMe_3_ (<24 h (Fig. 2B). Since the liquid DNP experiments were performed in glycerol (see below), the persistence of 5d in glycerol was also investigated (Fig. 2C); it showed a gradual degradation with an estimated half-life of ∼8 days. No detectable decrease in the DNP enhancement (see below) was observed during measurements at 315 K over a period of 4 h.

**Fig. 2:**
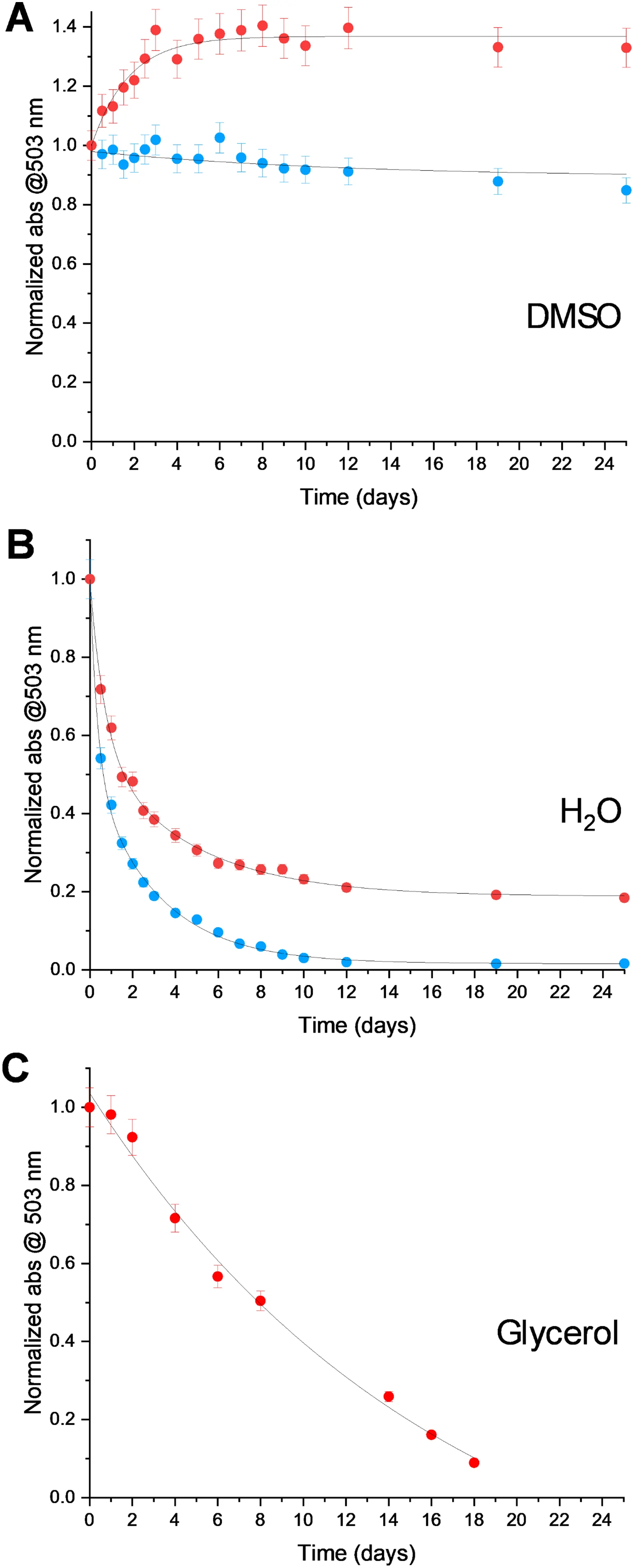
Persistence of the trimethyl derivative of trialkylammonium BDPA (BDPA-NMe_3_) (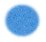) and BDPA-dendrimer 5d (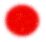) in DMSO (A) and H_2_O (B), as well as 5d in glycerol (C) at 23 °C, monitored by UV-Vis spectroscopy.^39^ The concentration of the radicals were 10 mM by weight, but the absolute radical concentration, determined by spin-counting was 7.5 mM and 5.6 mM for BDPA-NMe_3_ and BDPA-dendrimer 5d, respectively.

As mentioned above, DNP NMR in viscous liquids is emerging as a valuable approach for solution-state NMR studies at high magnetic fields and room temperature.^35^ When molecular tumbling of an analyte in solution is reduced, for example in viscous solvents,^30,35^ electron-nuclear dipolar interactions slow down, which allows efficient polarization transfer and sizable signal enhancements.^36^ Such polarization can also be observed for large molecules (with a long rotational correlation time) in aqueous solutions.^29,37^ Carbon-based radicals like BDPA-NMe_3_ and OX063 (Fig. 1) give enhancements through the SE pathway using this approach, correlating with molecular weight, with OX063 (*M*_W_ = 1360.8) showing the highest DNP performance.^30^ Given the high molecular weight of BDPA-dendrimer 5d (*M*_W_ = 4157.2), we investigated its DNP performance. At 9.4 T and 315 K, 5d produced a ^1^H DNP enhancement of 40 ± 5 at a concentration of 20 mM in glycerol (Fig. 3), a roughly twofold increase in DNP enhancement relative to OX063 (*ε* = 20 ± 3, 20 mM).^30^ DNP performance was also evaluated at radical concentrations of 10 and 40 mM for 5d, giving DNP enhancements of 18 ± 3 and 57 ± 6, respectively (Table S1). Although high viscosity leads to an increase in transverse relaxation rates and subsequent line broadening, using a viscous solvent is not a requirement for the DNP mechanism itself. Efficient polarization of small molecules, like ATP, can be achieved at viscosities only six times that of water, while larger biomolecules (∼15 kDa) possess sufficiently long rotational correlation times to exhibit sizable enhancements in pure aqueous solutions.^37^ Glycerol was chosen here to enable a direct and quantitative comparison with the trityl-based radical OX063.^30^

**Fig. 3:**
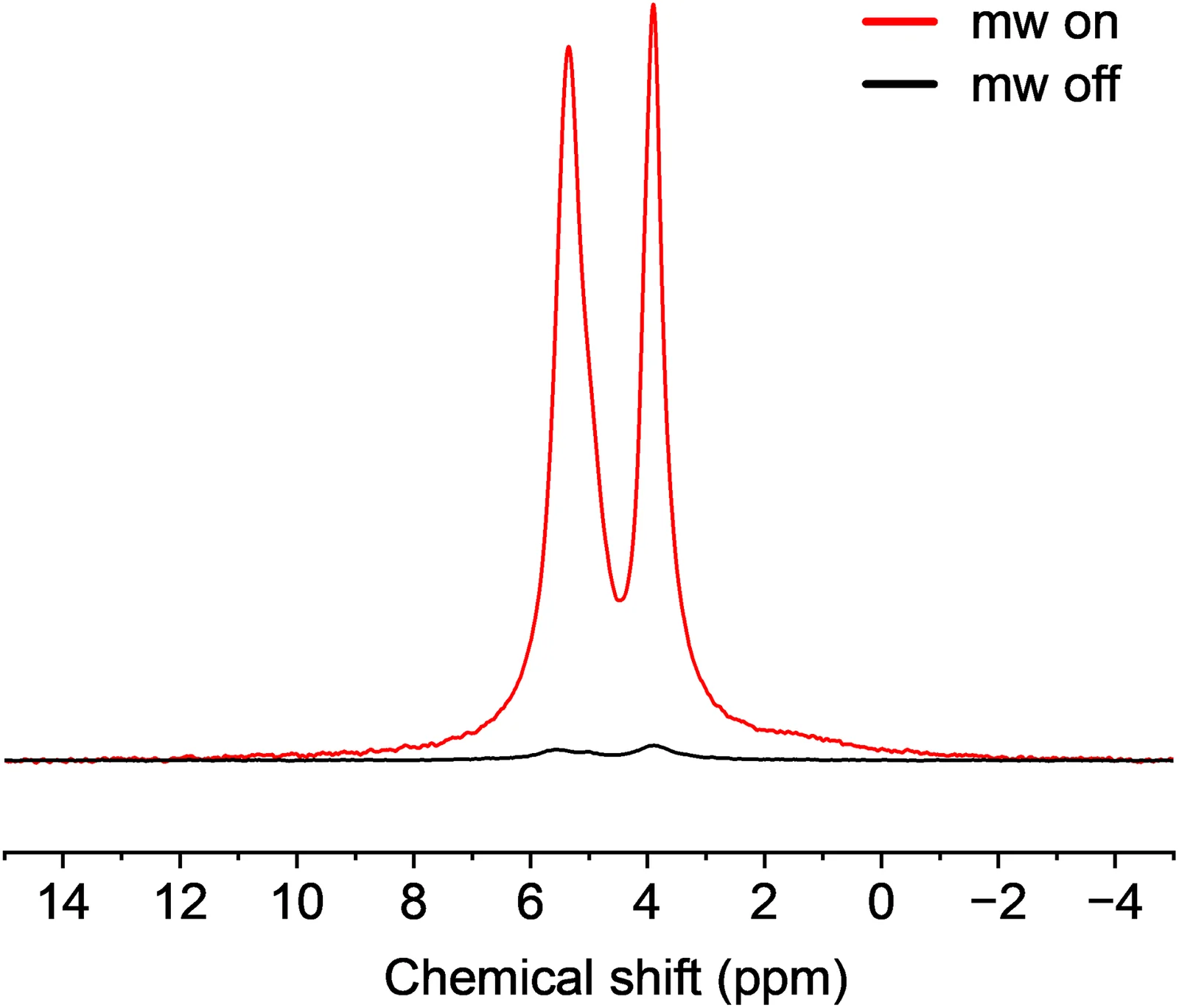
^1^H-DNP-enhanced NMR signal of glycerol with BDPA-dendrimer 5d at a concentration of 20 mM. Spectra were recorded at ∼315 K and 9.4 T with (red) and without (black) microwave irradiation at 263 GHz and 5 W of microwave power. The NMR signals are normalized with respect to the number of acquisitions.

In summary, we have developed a versatile click-based approach to prepare tetrasubstituted BDPA radicals, giving access to BDPA derivatives with tuneable size, solubility and stability. Among the four derivatives, BDPA-dendrimer 5d stood out, showing improved persistence in both water and DMSO. Importantly, 5d exhibited the best DNP performance reported thus far for ^1^H DNP NMR in viscous liquids, with enhancements of 57 at 40 mM, making the BDPA-dendrimer a promising polarizing agent for liquid-state DNP NMR.

## Conflicts of interest

There are no conflicts to declare.

## Supplementary Material

CC-062-D6CC01089J-s001

## Data Availability

Data for this article are available at Zenodo.org at https://doi.org/10.5281/zenodo.17975646. The supporting data has been provided as part of the supplementary information (SI). Supplementary information is available. See DOI: https://doi.org/10.1039/d6cc01089j.
