# Supplementary material for: Tetra-substituted BDPA radicals via click-chemistry and application to liquid-state DNP
Source: Chem Commun (Camb). 2026 May 13;62(42):10685–9. doi: 10.1039/d6cc01089j (PMC13169488; doi:10.1039/d6cc01089j)
Supplement: CC-062-D6CC01089J-s001 [file CC-062-D6CC01089J-s001.pdf]

Supplementary Information (SI) for ChemComm.
This journal is © The Royal Society of Chemistry 2026

Supporting Information

Tetra-substituted BDPA radicals via click-chemistry and application to liquid-state DNP

Iram M. Ahmad,[a] Pralambika Roy,[a] Andrei Kuzhelev,[b] Snorri Th. Sigurdsson*[a]

[a] *Department of Chemistry, Science Institute, University of Iceland, 107 Reykjavik, Iceland*
[b] *Institute of Physical and Theoretical Chemistry and Center for Biomolecular Magnetic Resonance (BMRZ), Goethe University, 60438 Frankfurt am Main, Germany*

Table of Contents

List of abbreviations 3
Synthetic procedure 4
General materials and methods 4
BDPA tetraazide **2** 5
BDPA-OH **4a** 8
BDPA-hydroxyamide **4b** 11
BDPA-glucose **4c** 15
BDPA-dendrimer **4d** 19
BDPA-sulfate **8** 23
BDPA-OH radical **5a** 26
BDPA-hydroxyamide radical **5b** 28
BDPA-glucose radical **5c** 30
BDPA-dendrimer radical **5d** 32
BDPA-sulfate radical **7** 34
Quantification of BDPA radicals 36
Persistence of BDPA-dendrimer **5d** 36
Solvent-dependent persistence 36
Liquid DNP NMR measurements 39
Experimental setup 39
^{1}H DNP Field Profile 40
DNP measurements at different concentrations of **5d** 41
References 41

List of Figures

Figure S1. ^{1}H-NMR spectrum of compound BDPA tetraazide **2**. 6

Figure S2. ^{13}C-NMR spectrum of BDPA tetraazide **2**. 6

Figure S3. IR spectrum of BDPA tetraazide **2**. 7

Figure S4. ^{1}H-NMR spectrum of compound **4a**. 9

Figure S5. ^{13}C-NMR spectrum of compound **4a**. 9

Figure S6. IR spectrum of compound **4a**. 10

Figure S7. ^{1}H-NMR spectrufm of compound **4b**. 13

Figure S8. ^{13}C-NMR spectrum of compound **4b**. 13

Figure S9. IR spectrum of compound **4b**. 14

Figure S10. ^{1}H-NMR spectrum of compound **4c**. 17

Figure S11. ^{13}C-NMR spectrum of compound **4c**. 17

Figure S12. IR spectrum of compound **4c**. 18

Figure S13. ^{1}H-NMR spectrum of compound **4d**. 21

Figure S14. ^{13}C-NMR spectrum of compound **4d**. 21

Figure S15. IR spectrum of compound **4d**. 22

Figure S16. ^{1}H NMR spectrum of compound **8**. 24

Figure S17. ^{13}C NMR spectrum of compound **8**. 24

Figure S18. IR spectrum of compound **8**. 25

Figure S19. IR spectrum of compound **5a**. 27

Figure S20. IR spectrum of compound **5b**. 29

Figure S21. IR spectrum of compound **5c**. 31

Figure S22. IR spectrum of compound **5d**. 33

Figure S23. IR spectrum of compound **7**. 35

Figure S24. The UV-vis absorbance spectra of **5d** (left) and its corresponding normalized absorbance at 503 nm (right), plotted as a function of time in DMSO (**A**), H_2O (**B**) and glycerol (**C**). 37

Figure S25. Comparison of persistence of filtered (■) and unfiltered (■) **5d** solution in DMSO (10 mM) at 23 °C, monitored by UV-vis spectroscopy at 503 nm. 38

Figure S26. Persistence of 5d for 1 mM (■), 5mM (■), 10 mM (■) and 20 mM (■) solutions in DMSO at 23 °C monitored by UV-vis spectroscopy at 503 nm. 38

Figure S27. **A**. The UV-vis absorbance of BDPA-dendrimer **4d** in DMSO over 7 days at 23 °C. **B**. Comparison of effect of DMSO on radical **5d** (■) with its non-radical **4d**. Anion formed in non-radical is shown by (▲) while the radical formed over time in the **4d** is indicated by (●). 39

Figure S28. Normalized absorbance of a 10 mM solution of **4d** in DMSO, in the presence (■) and absence of O_2 (■) at 503 nm plotted as a function of time. 39

Figure S29. X-band and J-band EPR of BDPA-dendrimer. **A**. X-band CW EPR spectrum of 20 mM **5d** in glycerol at 298 K. **B**. J-band CW EPR of 20 mM **5d** in glycerol at 298 K. **C.** J-band ED EPR of 0.3 mM **5d** at 100 K. 40

Figure S30. Field profile of the ^{1}H DNP enhancement for a solution of BDPA-dendrimer **5d** (20 mM) in glycerol at 9.4T and 315 K. 41

List of abbreviations

Aq.	Aqueous
ATR	Attenuated Total Reflection
BDPA	1,3- Bisdiphenylene-2- phenylallyl
Calcd.	Calculated
CuAAC	Cu-catalyzed azide-alkyne cycloaddition
CW	Continuous wave
DMF	*N,N*-dimethylformamide
DMSO	Dimethylsulfoxide
DNP	Dynamic nuclear polarization
EPR	Electron paramagnetic resonance
ESI	Electrospray ionization
HPLC	High-performance liquid chromatography
HRMS	High resolution mass spectrometry
NMR	Nuclear magnetic resonance
Pet-ether	Petroleum ether
ppm	Parts per million
RP	Reverse phase
Satd.	Saturated
R_f	Retention factor
TLC	Thin layer chromatography

Synthetic procedure

General materials and methods

All commercially available reagents were purchased from Sigma-Aldrich Co., abcr GmbH, and Polymer Factory, and were used without further purification. Polyester-8-hydroxyl-1-acetylene bis-MPA dendron, Gen-3 (**3d**) was purchased from Polymer Factory. All moisture sensitive reactions were carried out in oven-dried glassware under an inert atmosphere of Ar. CH_2Cl_2 was dried over calcium hydride and freshly distilled before use. Reagent grade DMSO and DMF were dried over molecular sieves (3 Å). Thin layer chromatography (TLC) was carried out using glass plates pre-coated with silica gel (Kieselgel 60 F_{254}, 0.2 mm, Silicycle) and compounds were visualized using UV light. Column chromatography was performed using 230–400 mesh silica gel (Silicycle). ^{1}H- and ^{13}C-NMR spectra were recorded at the frequencies stated, using deuterated solvents as internal standards on a Bruker Avance 400 MHz and 600 MHz spectrometers. Radicals show broadening and loss of NMR signals due to their paramagnetic nature and therefore, those NMR spectra are not shown. Mass spectrometric analyses of all organic compounds were performed on an ESI-HRMS (Bruker, MicrOTOF-Q). EPR spectra were recorded on a MiniScope MS200 with following experimental parameters: 9.43 GHz, microwave power 1 mW, sweep width 12 mT, modulation 0.2 mT, 23 °C. Purity of all radicals was analysed on an analytical Agilent 1200 HPLC system using a NUCLEODUR C18 Pyramid 4.6 x 150 mm analytical column with UV detection at λ = 254 nm. Solvent gradients for analytical RP-HPLC were run at 1.0 mL/min using the following gradient: solvent A, 0.1% TFA in H_2O; solvent B, CH_3CN; 0-2 min isocratic 0% B, 10 min linear gradient to 100% B, 2 min linear gradient to 0% B, 2 min isocratic 0% B. The UV-Vis spectra were recorded on an Agilent Cary UV-Vis Multicell Peltier spectrophotometer. The IR spectra were recorded on a Thermo Scientific Nicolet iS50-ATR IR spectrometer.

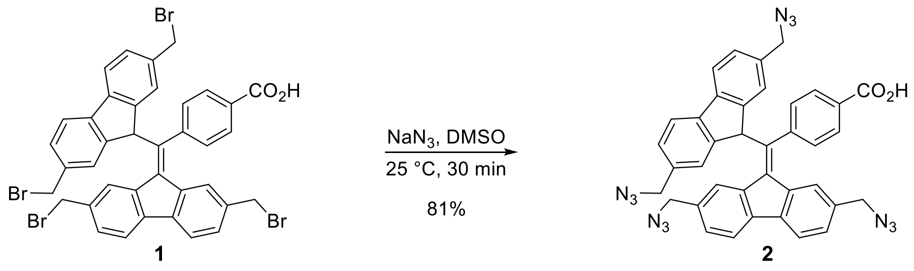

BDPA tetraazide 2. To a solution of compound **1**[1] (500 mg, 0.6 mmol) in DMSO (6 mL), was added NaN_3 (194.8 mg, 3.0 mmol) and the resulting solution was stirred at 23 °C for 30 min. The reaction was acidified with aq. HCl (1 N, 10 mL) and extracted with EtOAc (3 x 10 mL). The combined organic layers were washed with brine (2 x 20 mL), dried over Na_2SO_4 and concentrated *in vacuo*. The crude product was purified by flash-column chromatography using a gradient elution (CH_2Cl_2:MeOH; 100:0 to 98:02) to give **2** (331 mg, 81%) as an orange solid.

R_f (**1**) = 0.5, R_f (**2**) = 0.4 (CH_2Cl_2/MeOH 98:02); ^{1}H NMR (400 MHz, DMSO-d_6) δ 8.62 (s, 1H), 8.08 (d, *J* = 7.8 Hz, 1H), 7.93 (d, *J* = 7.8 Hz, 1H), 7.84 (d, *J* = 7.8 Hz, 2H), 7.71 (s, 2H), 7.56 (d, *J* = 8.2 Hz, 3H), 7.41 (dd, *J* = 7.8 Hz, 1.1 Hz, 2H), 7.25 (dd, *J* = 9.0 Hz, 1.1 Hz, 1H), 6.79 (d, *J* = 8.3 Hz, 2H), 6.62 (s, 1H), 5.61 (s, 1H), 4.56 (s, 2H), 4.46 (q, *J* = 7.8 Hz 2H), 4.02 (s, 2H) ppm; ^{13}C NMR (101 MHz, DMSO-d_6) δ 166.74, 144.38, 144.29, 142.35, 141.08, 140.12, 138.83, 138.48, 138.10, 135.33, 134.99, 134.76, 134.20, 129.81, 129.36, 128.73, 128.59, 128.44, 128.04, 126.37, 125.86, 124.52, 120.66, 119.97, 53.94, 53.71, 53.55, 51.89 ppm; IR (ATR, cm^{-1}): $\tilde{\nu}$ 2926.88 (s) (C-H), 2100 (vs) (N_3), 1694.06 (s) (C=O), 1417.13 (m) (C-C, aromatic) and 1240.41 (s) (C-O); HRMS (ESI): *m/z* calcd. for $C_{38}H_{26}N_{12}O_2+Na^+$ [M+Na$^+$] 705.2194, found 705.1864.

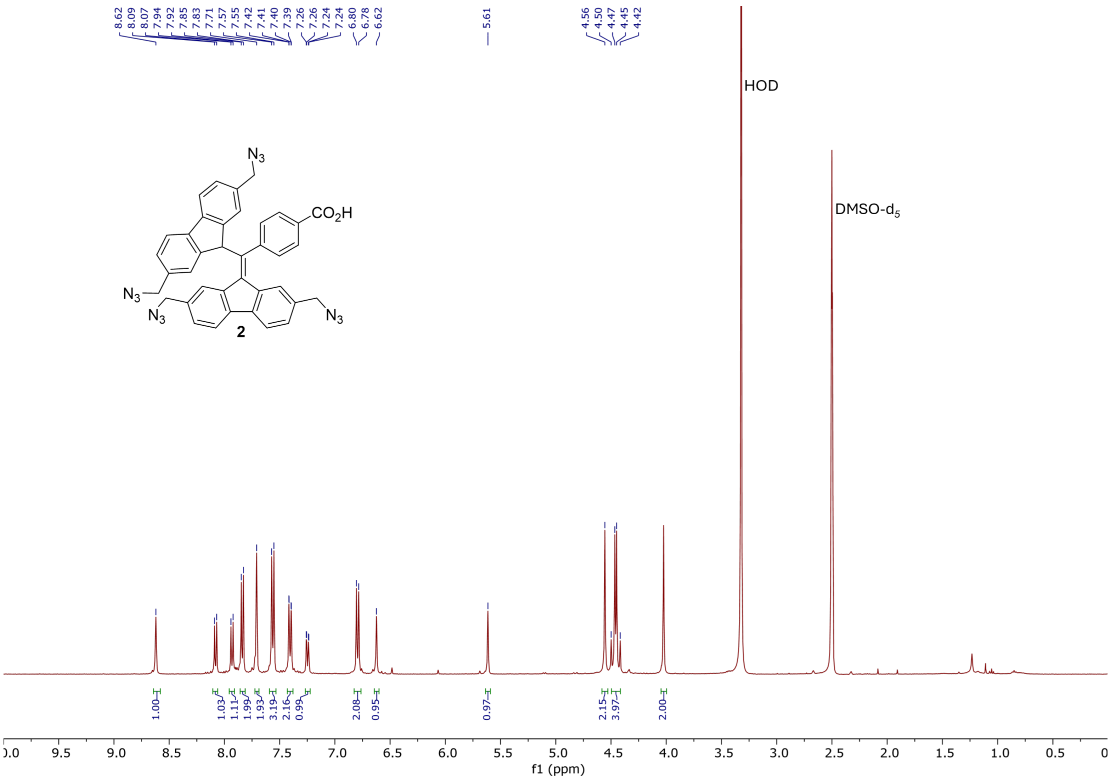

Figure S1. ^{1}H-NMR spectrum of compound BDPA tetraazide **2**.

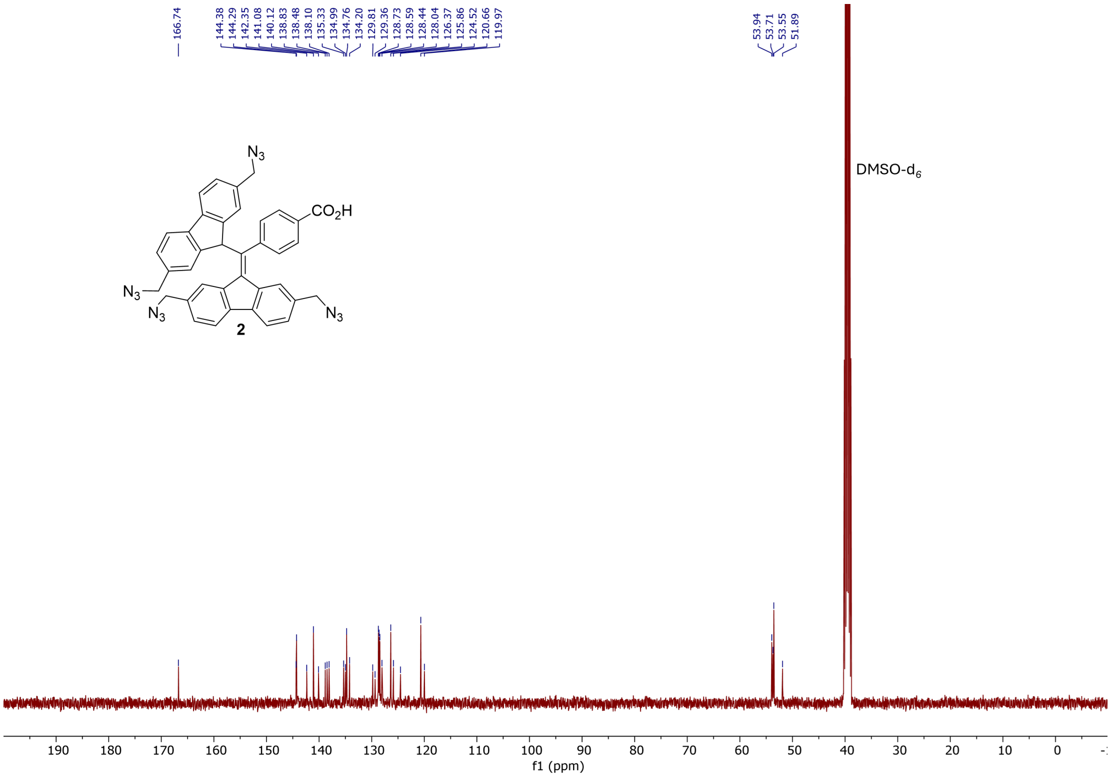

Figure S2. ^{13}C-NMR spectrum of BDPA tetraazide **2**.

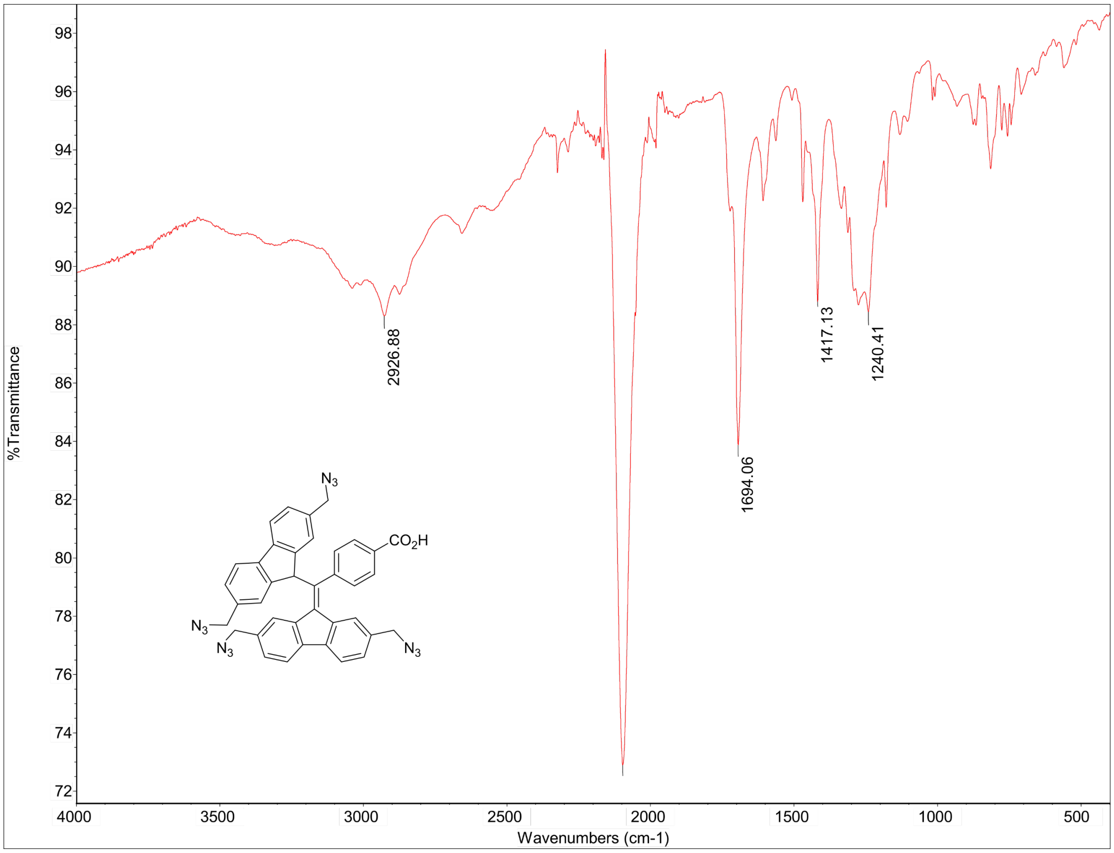

Figure S3. IR spectrum of BDPA tetraazide **2**.

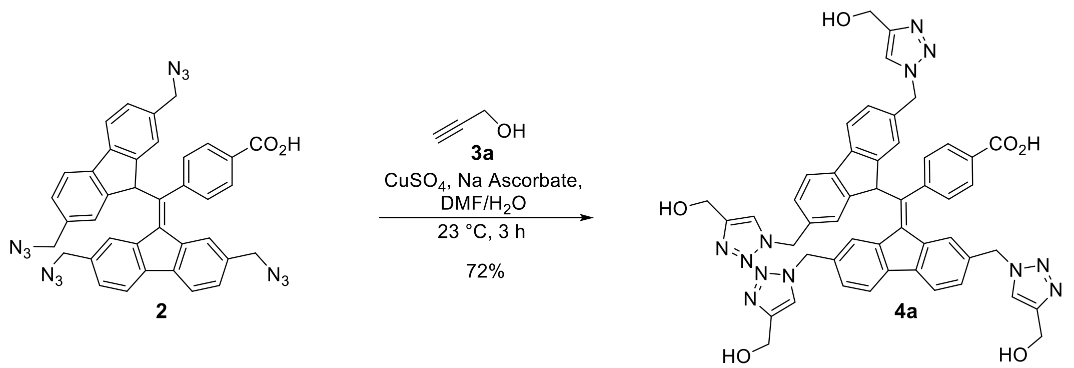

BDPA-OH 4a. To a solution of tetraazide **2** (60 mg, 0.088 mmol) in DMF (0.6 mL), was added a solution of sodium ascorbate (17.4 mg, 0.088 mmol) and $CuSO_4$ (4.4 mg, 0.018 mmol) in H_2O (0.2 mL) and the reaction mixture was stirred at 23 °C. After 5 min, propargyl alcohol **3a** (0.23 mL, 0.395 mmol) was added and the reaction mixture was stirred at 23 °C for 3h. The solvent was removed *in vacuo*, and the crude was washed with Et_2O (2 x 10 mL). The precipitate was collected by centrifugation, followed by decantation of the solvent. The precipitate was washed with H_2O (2 x 5 mL) and isolated again by centrifugation and decantation of the solvent. The precipitate was dissolved in DMSO (0.5 mL) and re-precipitated with Et_2O (10 mL). This step was repeated, and the precipitate was dried to give **4a** (57 mg, 72%) as a yellow solid.

^{1}H NMR (600 MHz, DMSO-d_6): δ 8.52 (s, 1H), 8.02 (d, J = 7.4 Hz, 2H), 7.97 (s, 2H), 7.90 (d, J = 7.9 Hz, 1H), 7.76 (d, J = 7.7 Hz, 2H), 7.63 (s, 2H), 7.50 (s, 1H), 7.47 (d, J = 7.9 Hz, 2H), 7.43 (d, J = 8.0 Hz, 1H), 7.32 (dd, J = 7.9, 1.5 Hz, 2H), 7.26 (dd, J = 7.9, 1.5 Hz, 1H), 6.59 (d, J = 7.9 Hz, 2H), 6.48 (s, 1H), 5.66 (s, 2H), 5.62 – 5.56 (m, 5H), 5.10 (s, 2H), 4.50 (s, 4H), 4.48 (s, 2H), 4.42 (s, 2H) ppm; ^{13}C NMR (151 MHz, DMSO-d_6): δ 148.28, 147.95, 144.44, 144.16, 140.81, 139.82, 138.81, 138.48, 138.04, 135.90, 135.30, 134.90, 134.47, 128.71, 128.49, 128.07, 128.04, 127.88, 125.97, 122.83, 122.60, 122.27, 120.55, 119.97, 55.00, 54.93, 54.82, 52.94, 52.84, 52.75, 51.88 ppm; IR (ATR, cm^{-1}): $\tilde{\nu}$ 3288.04 (s,b) (O-H, alcohol), 3145.06, 1654.88 (s) (C=O), 2930.58 (C-H), 1654.06 (s) (C=O), 1418.36 (m) (C-C, aromatic) and 1135.11 (s) (C-O); HRMS (ESI): *m/z* calcd. for $C_{50}H_{42}N_{12}O_6$+Na^+ [M+Na^+] 929.3242, found 929.3119.

HPLC:

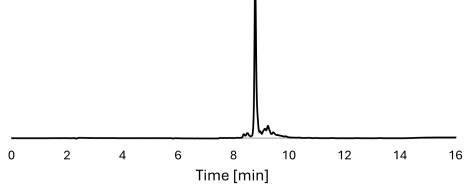

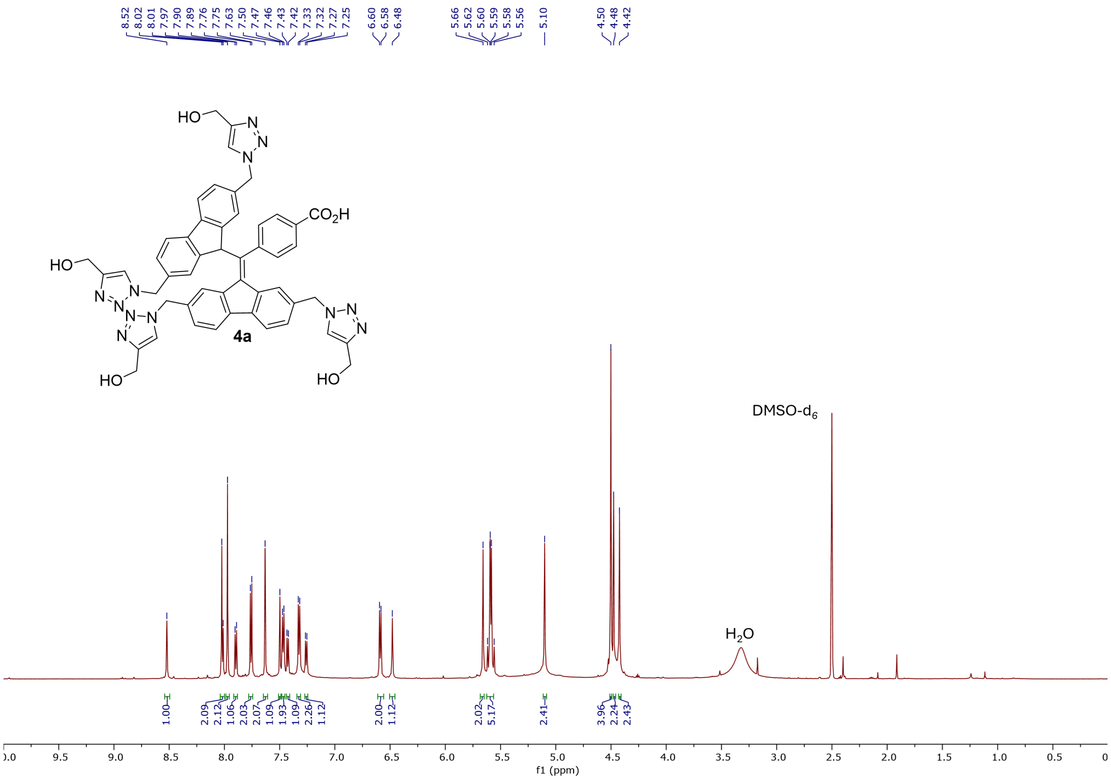

Figure S4. ^{1}H-NMR spectrum of compound **4a**.

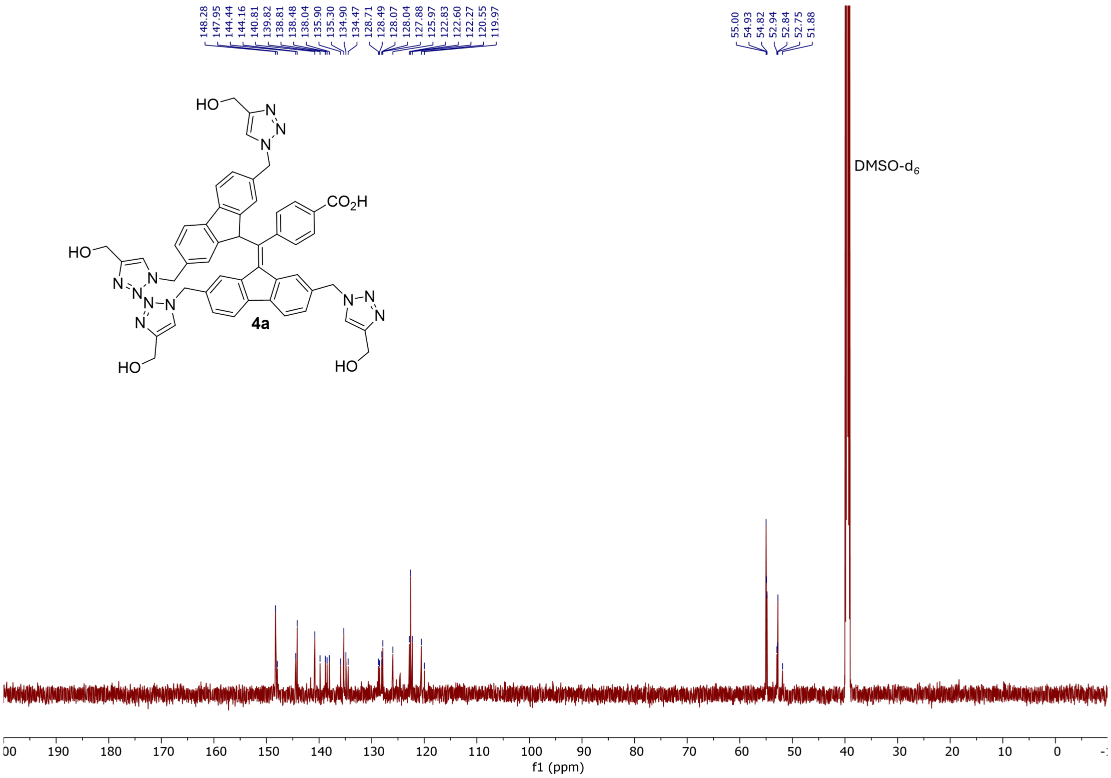

Figure S5. ^{13}C-NMR spectrum of compound **4a**.

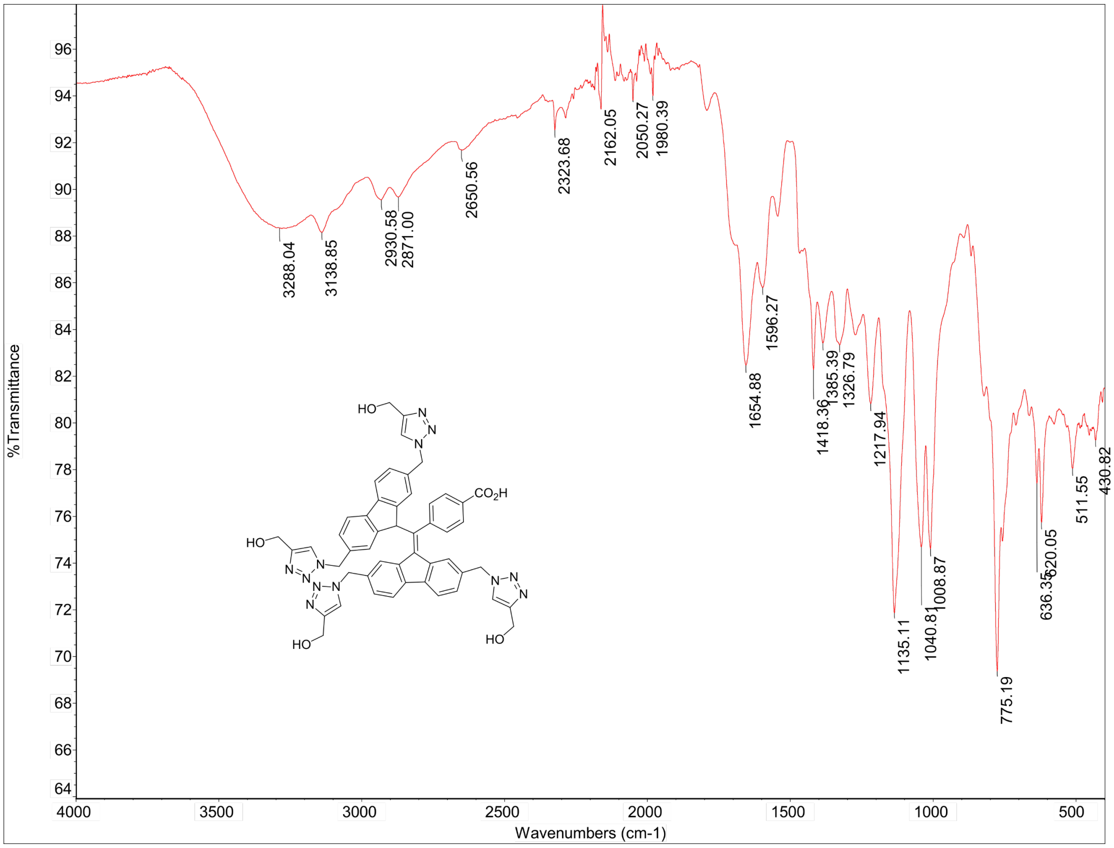

Figure S6. IR spectrum of compound **4a.**

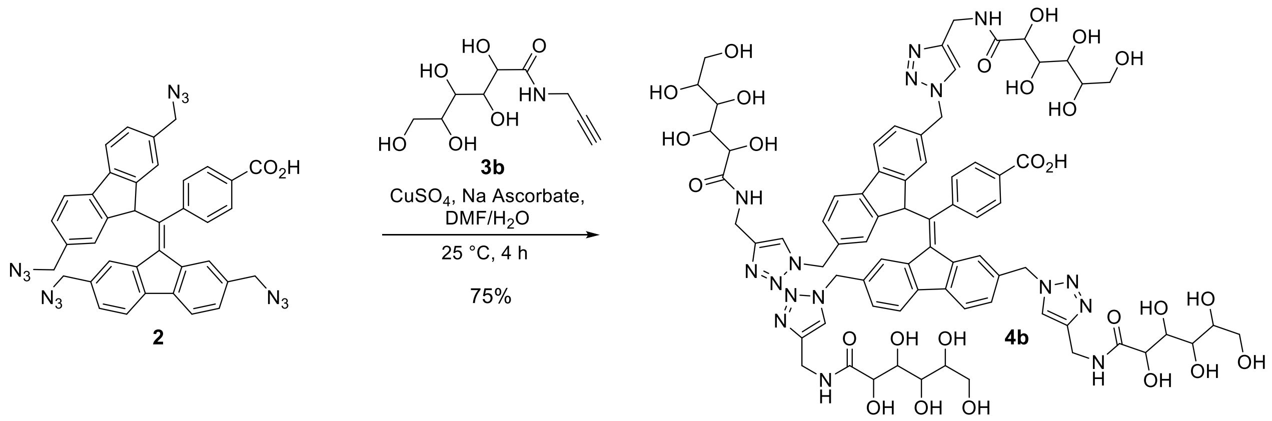

BDPA-hydroxyamide 4b. To a solution of tetraazide **2** (30 mg, 0.0439 mmol) in DMF (0.4 mL), was added a solution of sodium ascorbate (8.7 mg, 0.0439 mmol) and $CuSO_4$ (2.2 mg, 0.009 mmol) in H_2O (0.2 mL) at 23 °C. After 2 min a solution of *N*-2-propyn-1-yl-D-gluconamide **3b**[2] (40 mg, 0.198 mmol) in DMF (0.2 mL) was added and the reaction mixture was stirred at 23 °C for 4 h. The solvent was removed *in vacuo*, and the crude was washed with Et_2O (2 x 10 mL). The precipitate formed was collected by centrifugation, followed by decantation of the solvent. The precipitate was washed with H_2O (2 x 5 mL) and isolated again by centrifugation and decantation of the solvent. The precipitate was dissolved in 0.1% TFA H_2O: CH_3CN (7:1) and passed through C18 silica, followed by drying to afford **4b** (53 mg, 75%) as a brownish red solid.

^{1}H NMR (600 MHz, DMSO-d_6): δ 8.53 (s, 1H), 8.09-8.05 (m, , 3H), 8.03 – 7.98 (m, 3H), 7.95 (s, 2H), 7.89 (d, *J* = 7.8 Hz, 1H), 7.75 (d, *J* = 7.9 Hz, 2H), 7.63 (s, 2H), 7.55 (s, 1H), 7.48 (d, *J* = 7.9 Hz, 2H), 7.39 (d, *J* = 7.9 Hz, 1H), 7.29 (d, *J* = 7.9 Hz, 2H), 7.22 (d, *J* = 7.9 Hz, 1H), 6.63-6.61 (m, 2H), 6.49 (s, 1H), 5.66 (s, 2H), 5.61 – 5.56 (m, 5H), 5.38 (d, *J* = 4.7 Hz, 3H), 5.09 (s, 2H), 4.53 (s, 4H), 4.48 – 4.43 (m, 8H), 4.39-4.24 (m, 12H), 4.11 – 4.00 (m, 8H), 3.93 (s, 4H), 3.57 (d, *J* = 11.0 Hz, 4H), 3.48 (s, 9H) ppm; ^{13}C NMR (151 MHz, DMSO-d_6) δ 172.58, 166.83, 145.26, 145.22, 144.99, 144.41, 144.13, 141.90, 140.81, 139.84, 138.83, 138.46, 138.02, 135.84, 135.24, 134.91, 134.49, 134.42, 129.83, 128.74, 128.41, 128.17, 128.14, 127.89, 127.83, 126.03, 125.37, 124.52, 123.11, 122.93, 122.43, 120.64, 120.62, 120.58, 120.02, 118.03, 73.61, 72.25, 71.52, 70.16, 63.30, 52.99, 52.90, 52.82, 51.87 ppm; IR (ATR, cm^{-1}): $\tilde{\nu}$ 3296.48 (s,b) (O-H, alcohol), 2935.38 (C-H), 1647.66 (s) (C=O), 1418.83 (m) (C-C, aromatic) and 1123.61 (s) (C-O), 1051.19 (m) (C-N); HRMS (ESI): *m/z* calcd. for $C_{74}H_{86}N_{16}O_{26}$+2Na$^+$ [M+2Na$^+$] 830.2842, found 830.2794.

HPLC:

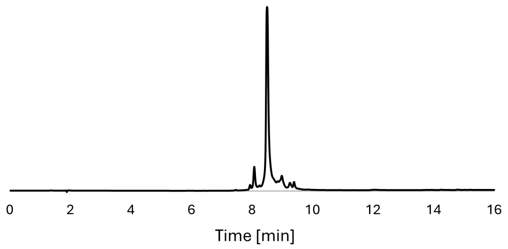

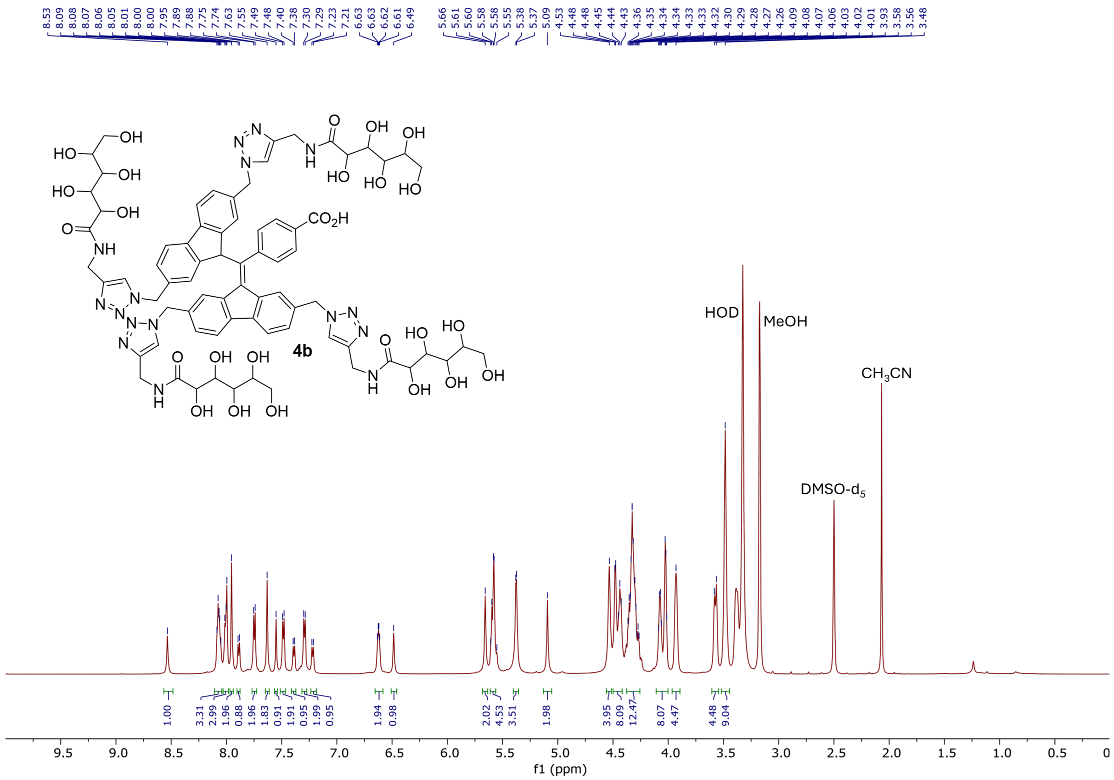

Figure S7. ^{1}H-NMR spectrufm of compound **4b**.

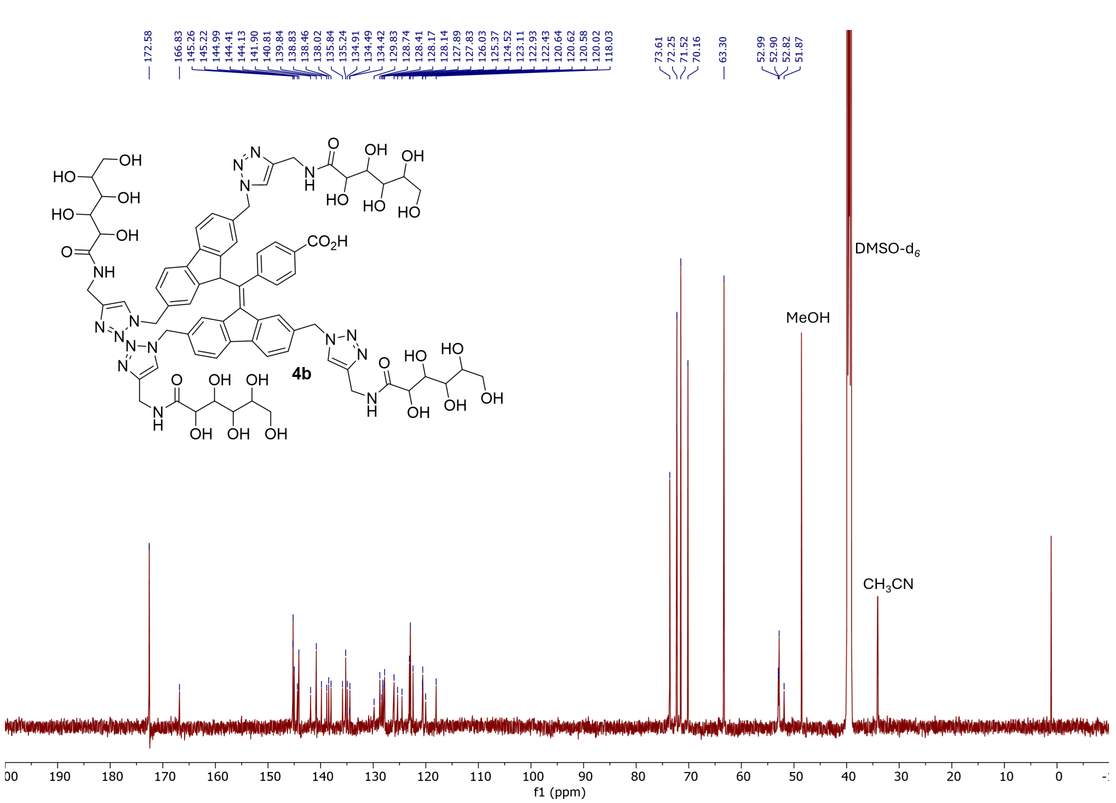

Figure S8. ^{13}C-NMR spectrum of compound **4b**.

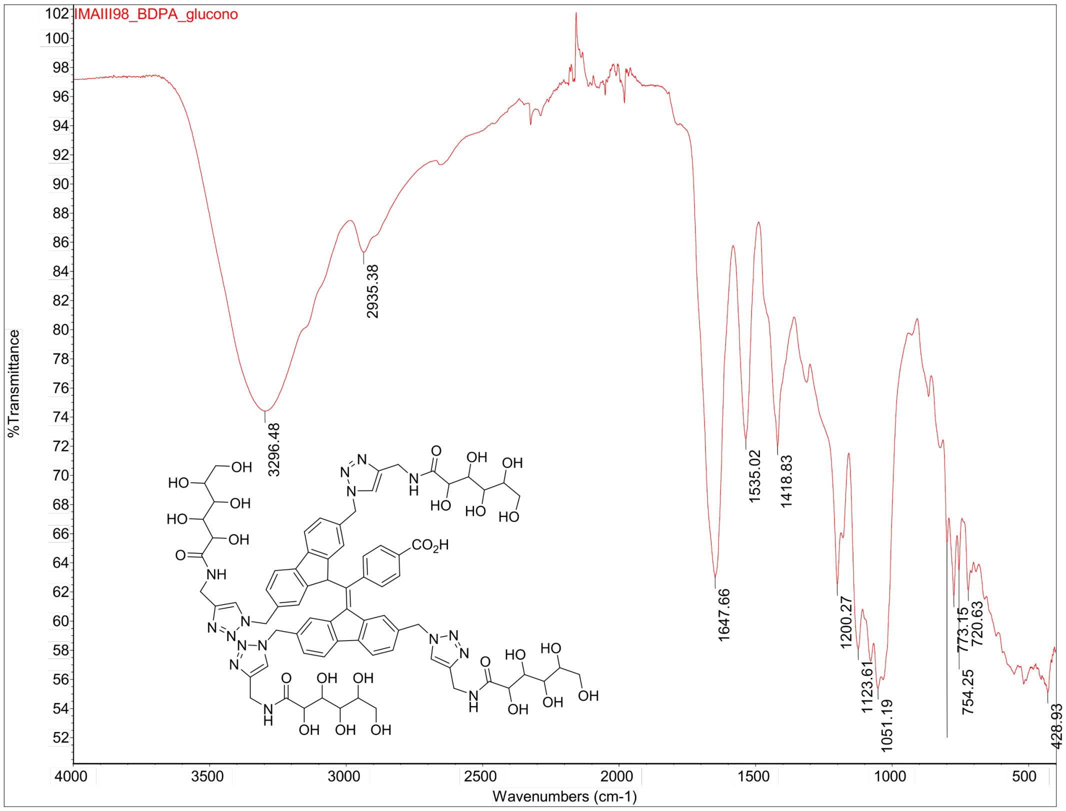

Figure S9. IR spectrum of compound **4b.**

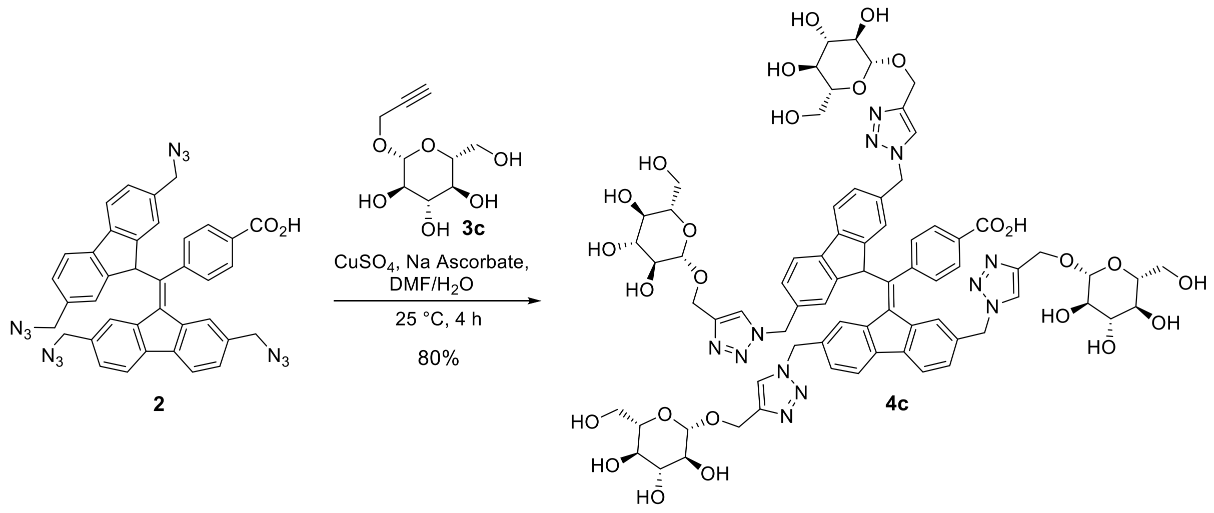

BDPA-glucose 4c. To a solution of tetraazide **2** (30 mg, 0.0439 mmol) in DMF (0.4 mL), was added a solution of sodium ascorbate (8.7 mg, 0.0439 mmol) and $CuSO_4$ (2.2 mg, 0.009 mmol) in H_2O (0.2 mL) at 23 °C. After 2 min a solution of 2-propyn-1-yl-β-D-glucopyranoside **3c**[3] (43.15 mg, 0.198 mmol) in DMF (0.2 mL) was added and the reaction mixture stirred at 23 °C for 4 h. The solvent was removed *in vacuo* and the crude was washed with Et_2O (20 mL). The precipitate was collected by centrifugation, followed by decantation of the solvent. The precipitate was dissolved in H_2O (0.25 mL) and re-precipitated with Et_2O (10 mL). The precipitate was dissolved in H_2O: CH_3CN (7:1) and passed through C18 silica, followed by drying *in vacuo* to afford **4c** (54.5 mg, 80%) as a reddish-orange solid.

^{1}H NMR (600 MHz, DMSO-d_6): δ 8.53 (s, 1H), 8.24 (s, 1H), 8.19 (s, 2H), 8.03 (d, *J* = 7.9 Hz, 1H), 7.90 (d, *J* = 7.8 Hz, 1H), 7.75 (dd, *J* = 7.9, 2.0 Hz, 2H), 7.73 (s, 1H), 7.63 (d, *J* = 7.3 Hz, 2H), 7.46-7.44 (m,, 3H), 7.32 (t, *J* = 7.9 Hz, 2H), 7.25 (d, *J* = 7.9 Hz, 1H), 6.64 – 6.59 (m, 2H), 6.49 (s, 1H), 5.69 (s, 2H), 5.69-5.58 (m, 6H), 5.14 (s, 2H), 4.85 – 4.81 (m, 5H), 4.77 (d, *J* = 12 Hz, 3H), 4.65 – 4.56 (m, 9H), 4.27 – 4.23 (m, 6H), 3.70-3.67 (m, 5H), 3.47 – 3.43 (m, 5H), 3.15-3.10 (m, 10H), 3.06-3.03 (m, 5H), 2.98-2.94 (m, 4H) ppm; ^{13}C NMR (151 MHz, DMSO-d_6) δ 166.69, 162.23, 144.29, 141.82, 140.77, 140.74, 139.80, 138.74, 138.38, 137.95, 135.64, 135.10, 135.08, 134.78, 134.33, 129.60, 128.66, 128.15, 128.08, 127.91, 127.81, 125.97, 125.34, 124.44, 124.01, 120.61, 120.52, 119.94, 102.20, 102.14, 102.12, 102.05, 76.82, 76.77, 76.59, 76.56, 73.27, 70.03, 70.00, 61.52, 61.44, 61.41, 61.25, 61.06, 61.04, 52.96, 52.86, 51.81 ppm; IR (ATR, cm^{-1}): $\tilde{\nu}$ 3297.29 (s,b) (O-H, alcohol), 2878.16 (C-H), 1652.92 (s) (C=O), 1050.19 (m) (C-O); HRMS (ESI): *m/z* calcd. for $C_{74}H_{82}N_{12}O_{26}+Na^+$ [M+Na^+] 1577.5355, found 1577.5250.

HPLC:

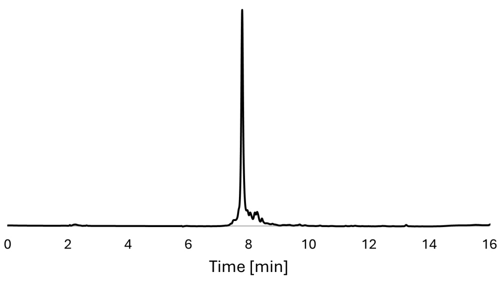

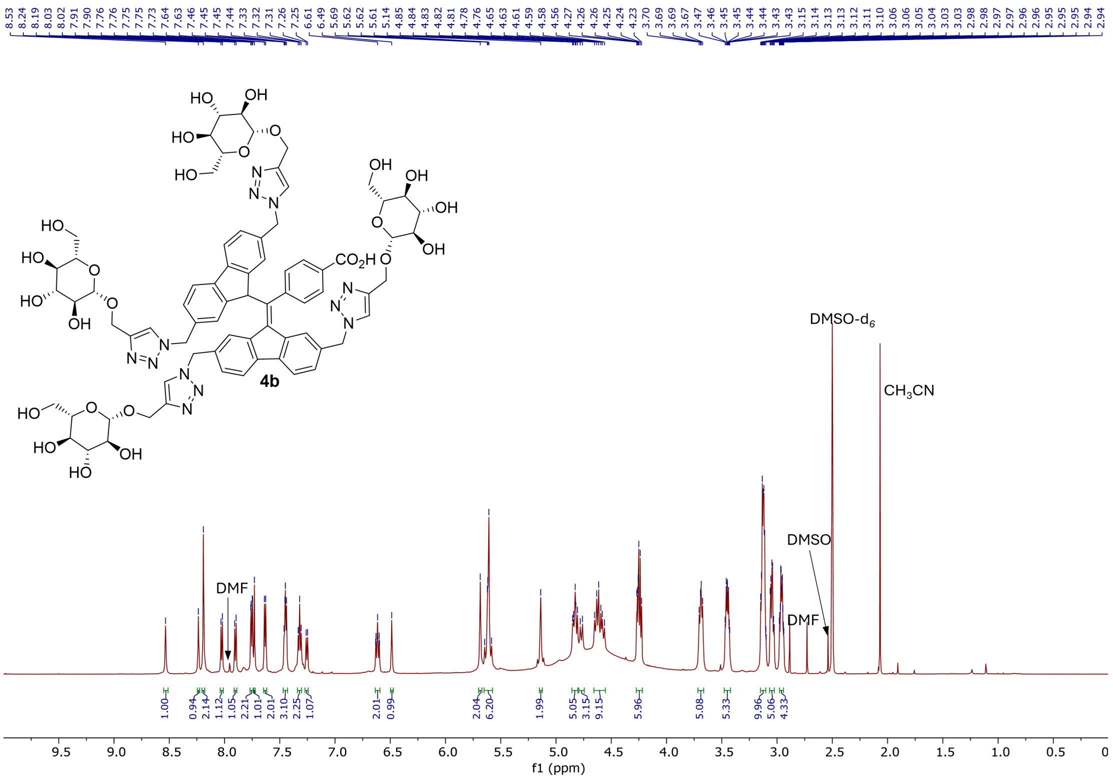

Figure S10. ^{1}H-NMR spectrum of compound **4c**.

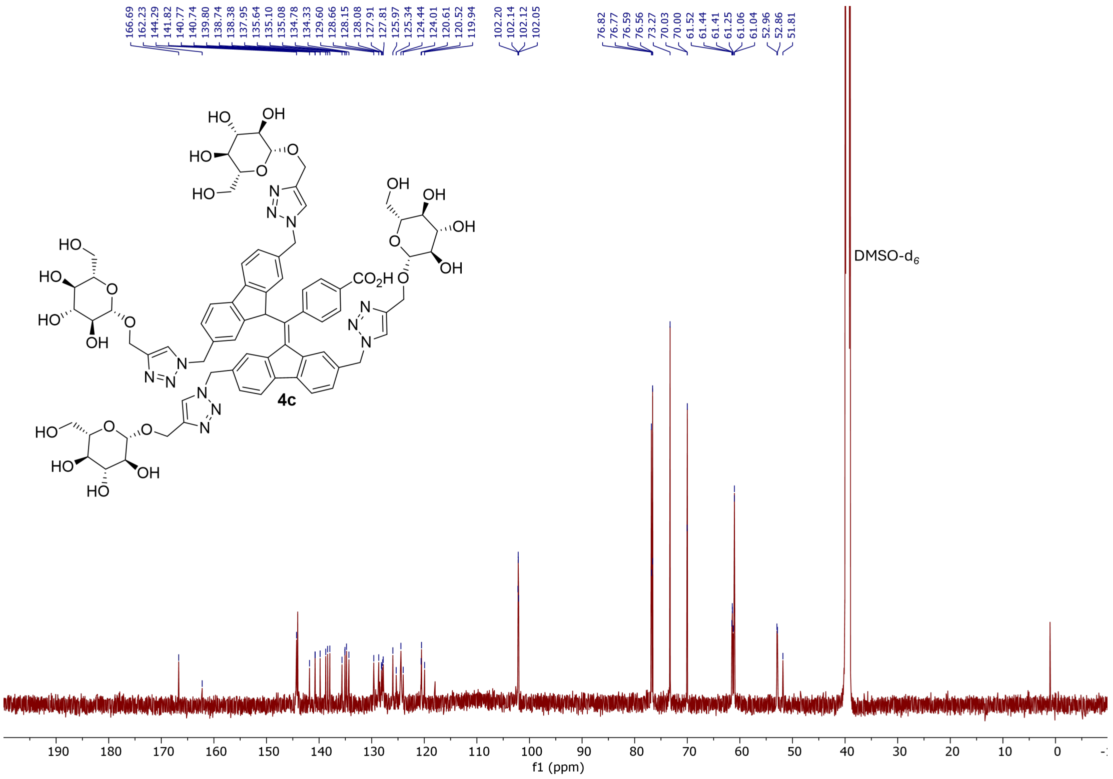

Figure S11. ^{13}C-NMR spectrum of compound **4c**.

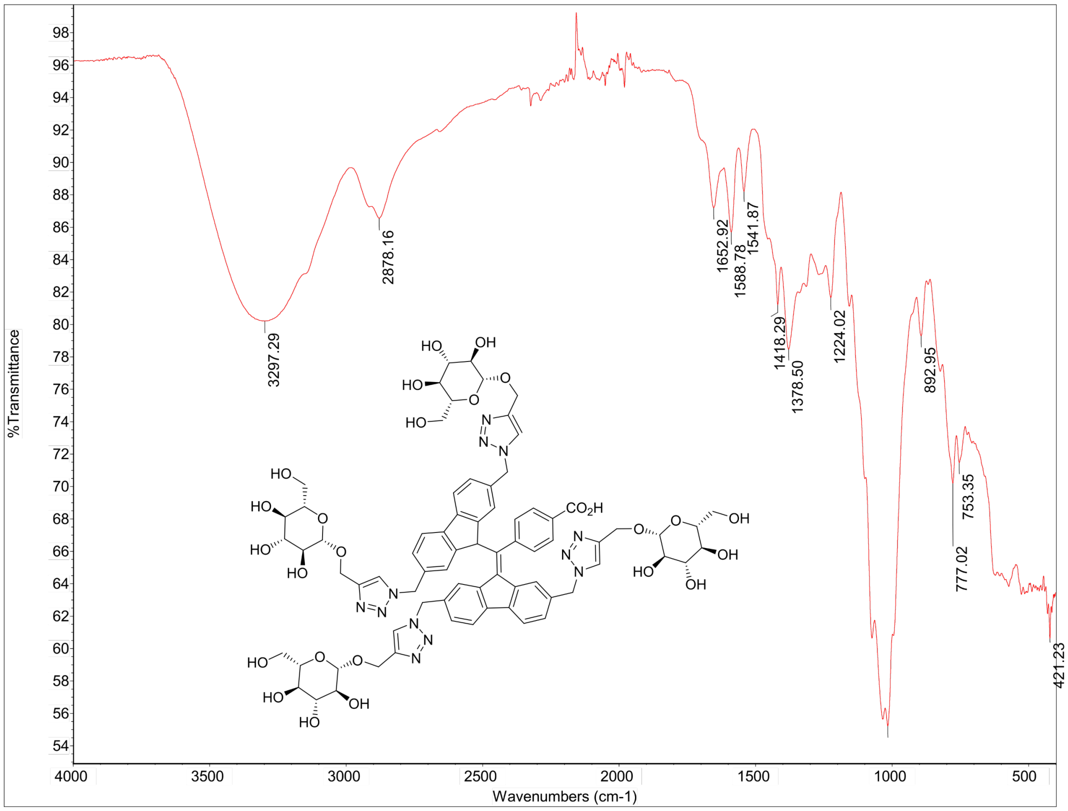

Figure S12. IR spectrum of compound **4c**.

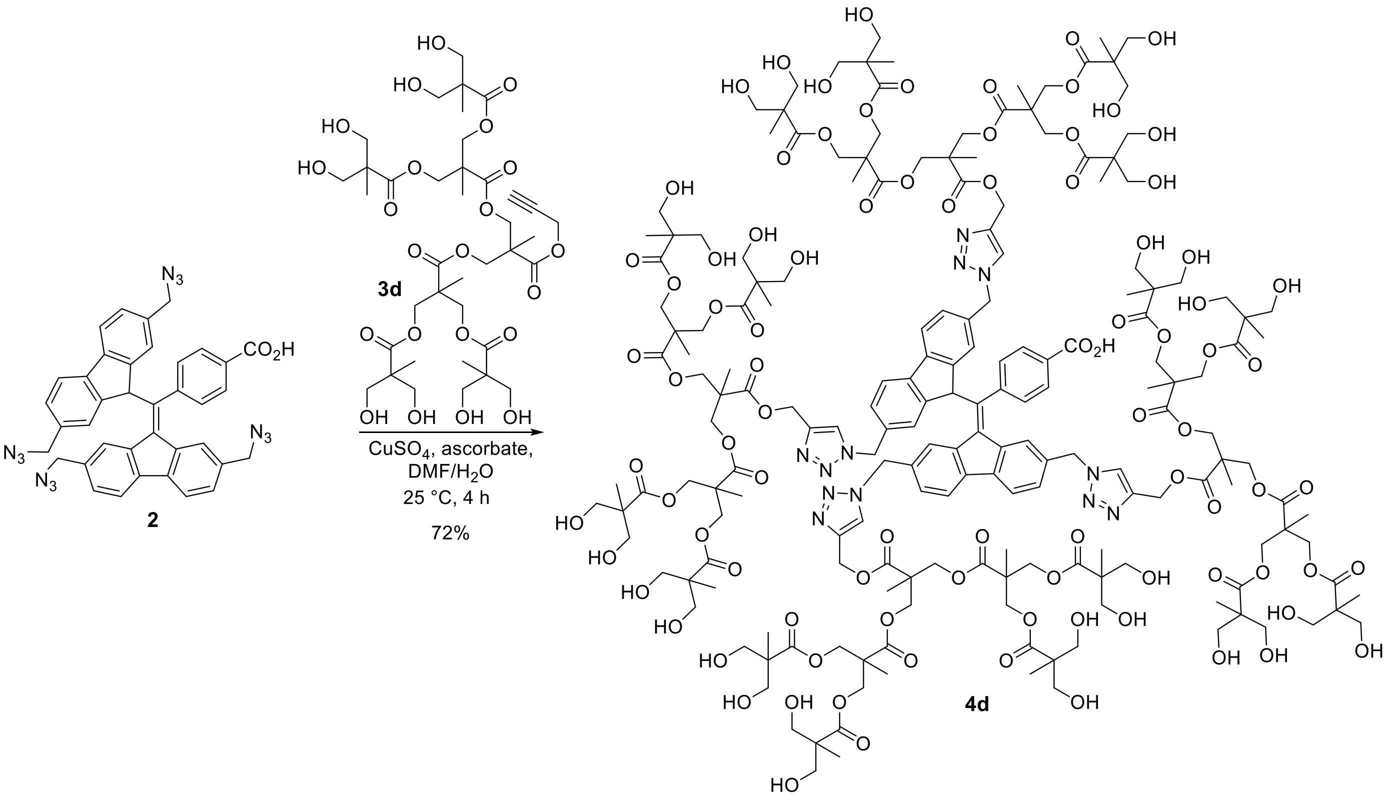

BDPA-dendrimer 4d. To a solution of compound **2** (20 mg, 0.029 mmol) in DMF (0.3 mL), was added a solution of sodium ascorbate (5.8 mg, 0.029 mmol) and $CuSO_4$ (1.5 mg, 0.0058 mmol) in H_2O (0.2 mL) at 23 °C. After 2 min a solution of alkyne dendrimer **3d** (112 mg, 0.1289 mmol) in DMF (0.3 mL) was added and the reaction mixture was stirred at 23 °C for 4 h. The solvent was removed *in vacuo,* and the crude was washed with Et_2O (20 x 2 mL). The reaction mixture was poured into Et_2O (10 mL), the precipitate collected by centrifugation, followed by decantation of the solvent. The precipitate was redissolved in MeOH (1 mL) and re-precipitated by addition of Et_2O (10 mL). The product was further purified by flash-column chromatography using a gradient elution (CH_2Cl_2:MeOH; 85:15 to 65:35) to give **4d** (1.08 g, 90%) as an orange solid.

R_f (**4d**) = 0.3 (CH_2Cl_2/ MeOH 7:3); ^{1}H NMR (400 MHz, Methanol-d_4): δ 8.26 (s, 1H), 8.18 (s, 1H), 8.10 (s, 2H), 8.00 (d, J = 7.9 Hz, 1H), 7.85 (d, J = 7.8 Hz, 1H), 7.77 (s, 1H), 7.74 (d, J = 2.3 Hz, 2H), 7.54 (t, J = 8.5 Hz, 3H), 7.48 (s, 2H), 7.38 (d, J = 7.9 Hz, 2H), 7.23 (d, J = 7.8 Hz, 1H), 6.55 (d, J = 8.1 Hz, 2H), 6.38 (s, 1H), 5.77 (d, J = 3.3 Hz, 3H), 5.73-5.64 (m, 5H), 5.32 (s, 2H), 5.27 (s, 4H), 5.19 (s, 2H), 5.09 (s, 2H), 4.34 – 4.15 (m, 63H), 3.71 – 3.65 (m, 40H), 3.62-3.57 (m, 32H), 1.31 – 1.28 (m, 10H), 1.26 – 1.19 (m, 39H), 1.14 (s, 33H), 1.13 (s, 10H).ppm; ^{13}C NMR (101 MHz, Methanol-d_4): δ 175.98, 175.95, 173.73, 173.69, 173.68, 173.63, 173.61, 146.97, 146.17, 143.85, 143.84, 143.44, 142.96, 141.98, 141.37, 140.73,

140.64, 140.32, 138.44, 136.54, 136.51, 136.13, 135.20, 132.36, 129.96, 129.48, 129.02, 128.98, 128.90, 126.88, 126.64, 126.54, 125.75, 121.95, 121.91, 121.16, 71.12, 69.19, 68.55, 67.21, 67.17, 66.88, 66.12, 65.78, 59.03, 58.94, 55.16, 54.96, 53.66, 51.78, 47.88, 47.86, 39.42, 33.04, 30.74, 25.98, 23.70, 19.51, 18.20, 18.16, 18.09, 18.04, 17.37, 15.43, 14.42 and 13.99 ppm; IR (ATR, cm^{-1}): $\tilde{\nu}$ 3369.88 (s,b) (O-H, alcohol), 2882.91 (C-H), 1723.61 (s) (C=O, ester), 1466.11 (m) (C-C), 1029.75 (m) (C-O); HRMS (ESI): *m/z* calcd. for $C_{190}H_{266}N_{12}O_{90}$ +4Na^+ [M+4Na^+] 1062.4060, found 1062.3909.

HPLC:

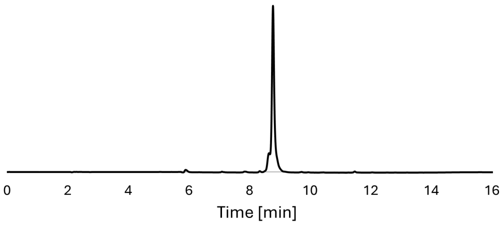

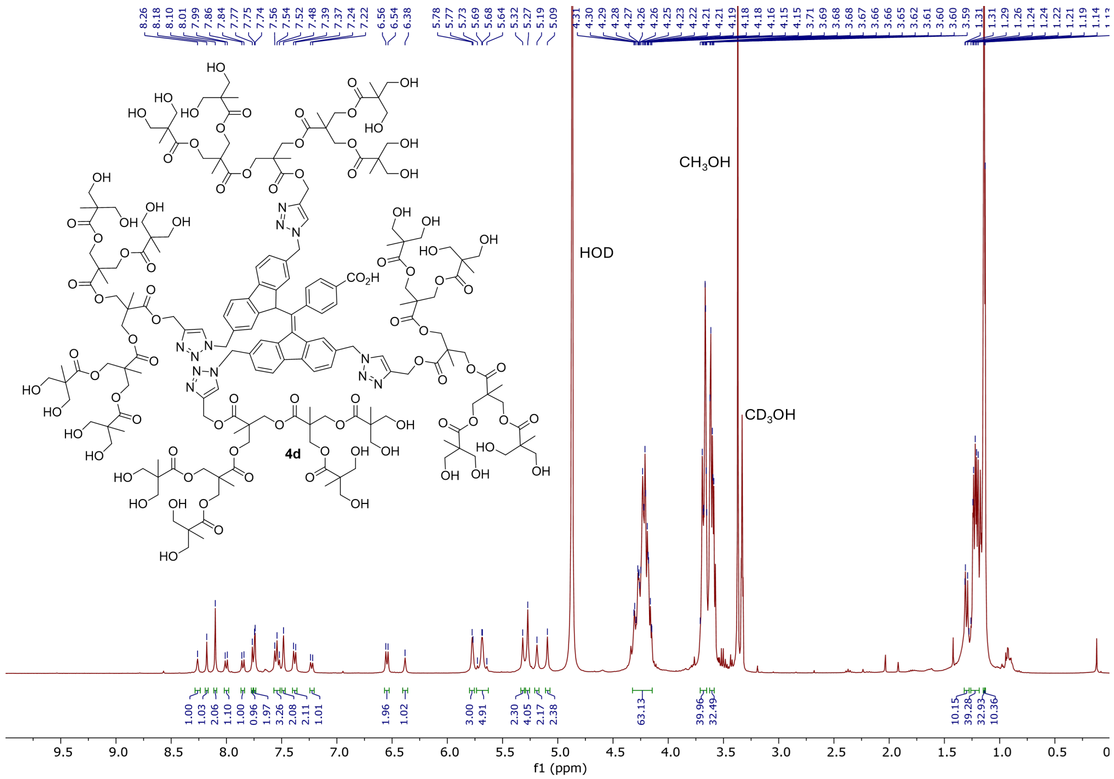

Figure S13. ^{1}H-NMR spectrum of compound **4d**.

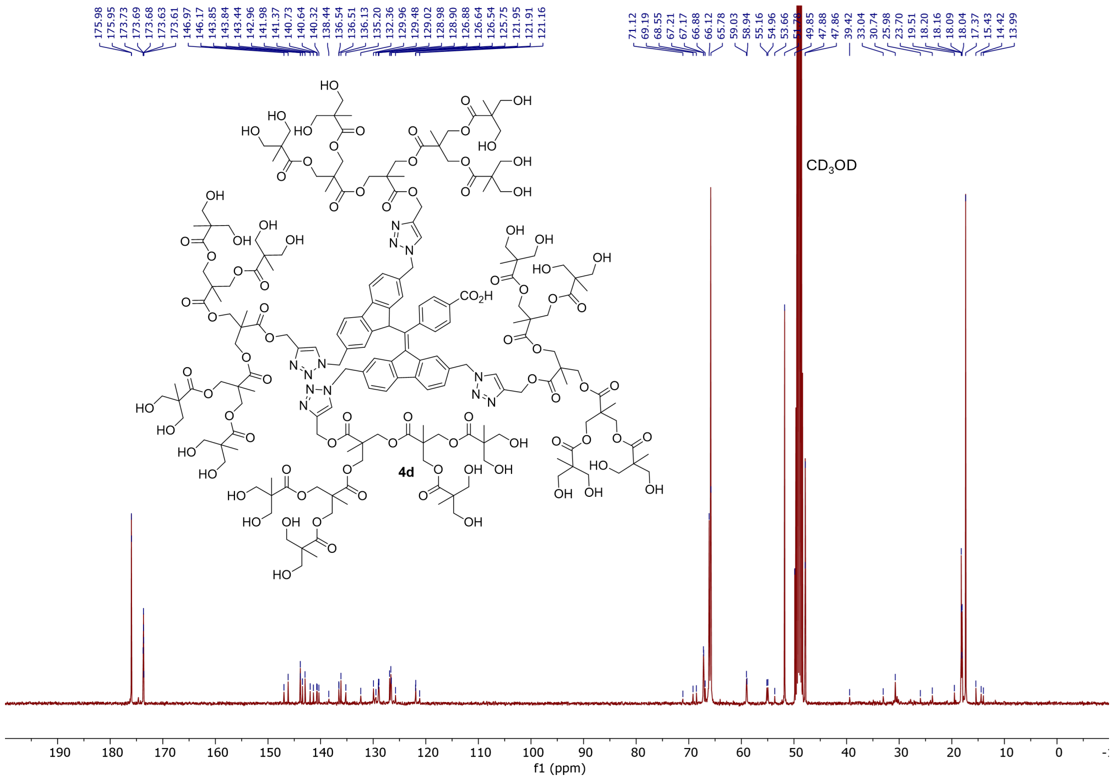

Figure S14. ^{13}C-NMR spectrum of compound **4d**.

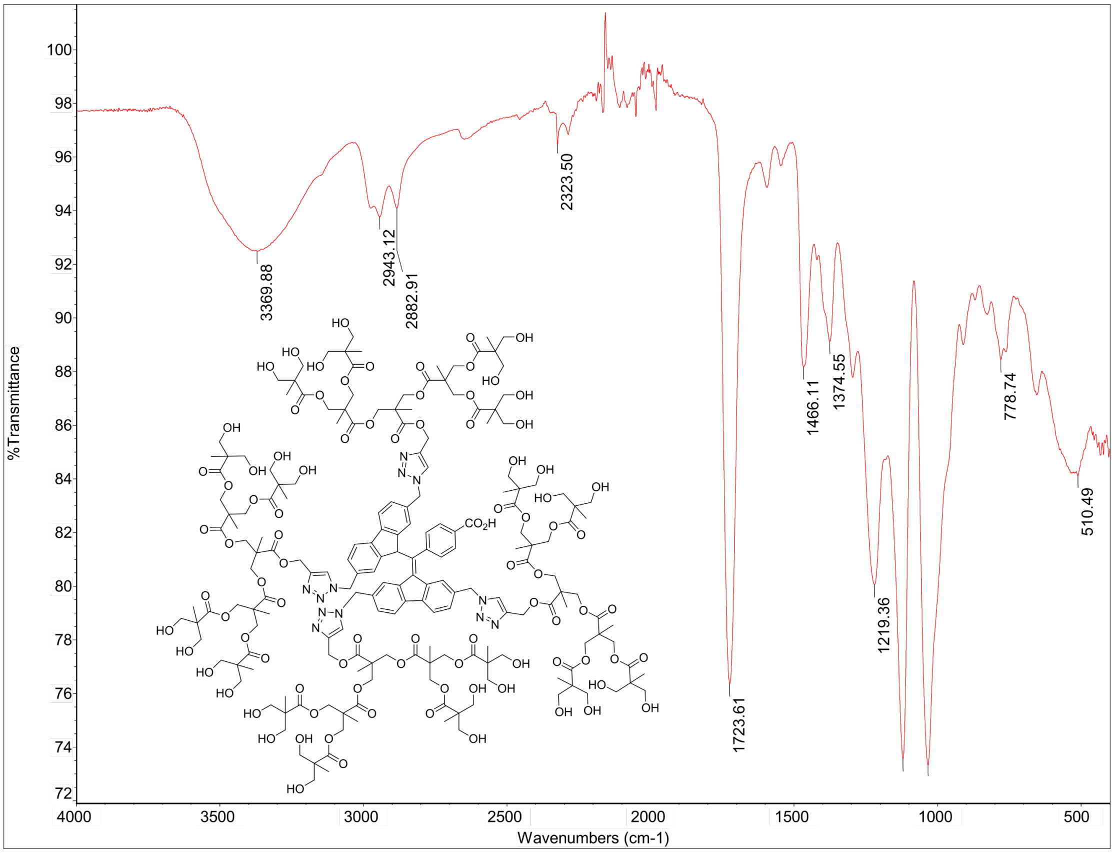

Figure S15. IR spectrum of compound **4d**.

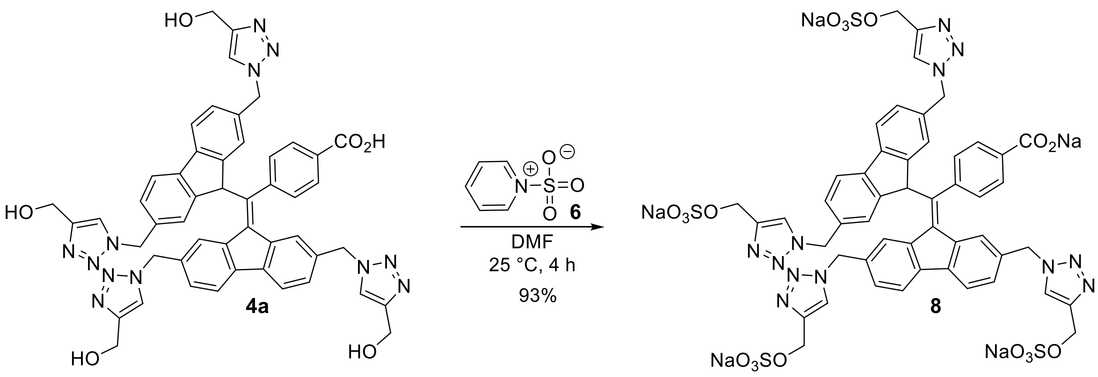

BDPA-sulfate 8. Sulfur trioxide pyridine complex **6** (28 mg, 0.176 mmol) was taken in an oven dried round bottom flask. To this, a solution of compound **4a** (20 mg, 0.022 mmol) in DMF (1.0 mL), was added and the resulting solution was stirred at 23 °C for 3 h. Satd. $NaHCO_3$ (0.5 mL) was added to the solution and stirred for 15 min. Water was evaporated *in vacuo* and the residue was precipitated using MeOH:Et_2O (1:10). The precipitate was dissolved in MeOH and filtered. The filtrate was dried and re-precipitated. The precipitate was dried in vacuo to give **8** (27 mg, 93%) as a blue solid.

^{1}H NMR (600 MHz, Methanol-d_4): δ 8.25 (s, 1H), 8.10 (s, 1H), 8.02 (s, 2H), 7.95 (d, *J* = 7.9 Hz, 1H), 7.80 (d, *J* = 7.8 Hz, 1H), 7.70 (d, *J* = 8.0 Hz, 2H), 7.67 (s, 1H), 7.56 (d, *J* = 8.1 Hz, 2H), 7.49 (d, *J* = 6.6 Hz, 3H), 7.31 (dd, *J* = 7.9, 1.6 Hz, 2H), 7.16 (dd, *J* = 7.9, 1.6 Hz, 1H), 6.57 (d, *J* = 8.0 Hz, 2H), 6.39 (s, 1H), 5.81 (d, *J* = 1.6 Hz, 1H), 5.73 (s, 2H), 5.64 (s, 4H), 5.15 (s, 2H), 5.11 (s, 2H), 5.08 (s, 4H), 4.94 (s, 2H) ppm;^{13}C NMR (151 MHz, Methanol-d_4): δ 174.94, 161.49, 146.94, 146.18, 144.97, 144.87, 144.60, 142.94, 141.98, 141.38, 140.78, 140.65, 140.31, 138.56, 136.57, 136.14, 135.02, 129.97, 129.42, 128.91, 128.80, 128.73, 126.84, 126.56, 126.02, 125.81, 125.72, 125.65, 121.72, 120.91, 61.80, 61.73, 61.67, 55.08, 54.88, 53.67 ppm; IR (ATR, cm^{-1}): $\tilde{\nu}$ 3409.33 (s,b) (O-H, alcohol), 2882.91 (C-H), 1599.84 (s) (C-C), 1382.67 (s) (S=O), 1250.11 (s) (C-N), 1050.19 (m) (C-O); HRMS (ESI): calcd. for $C_{50}H_{37}N_{12}O_{18}S_4Na$ [M^{4-}] 305.5333, found 305.5472.

<u>HPLC</u>:

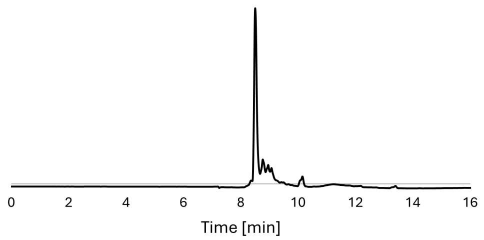

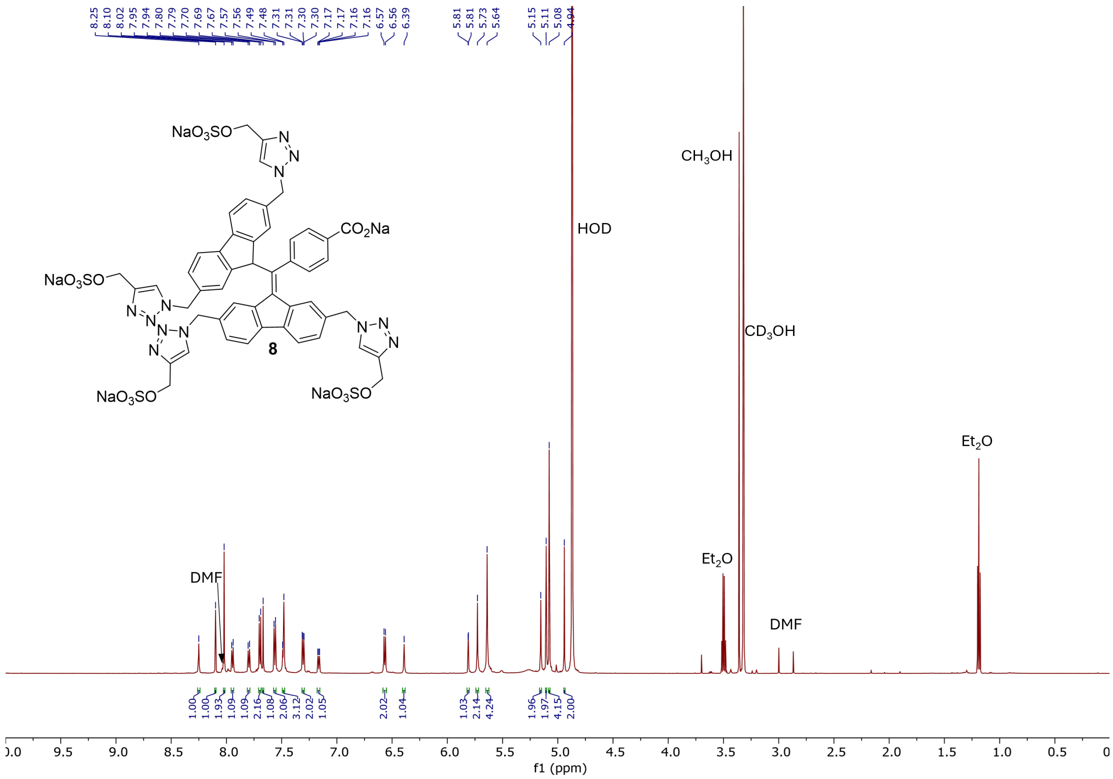

Figure S16. ^{1}H NMR spectrum of compound **8**.

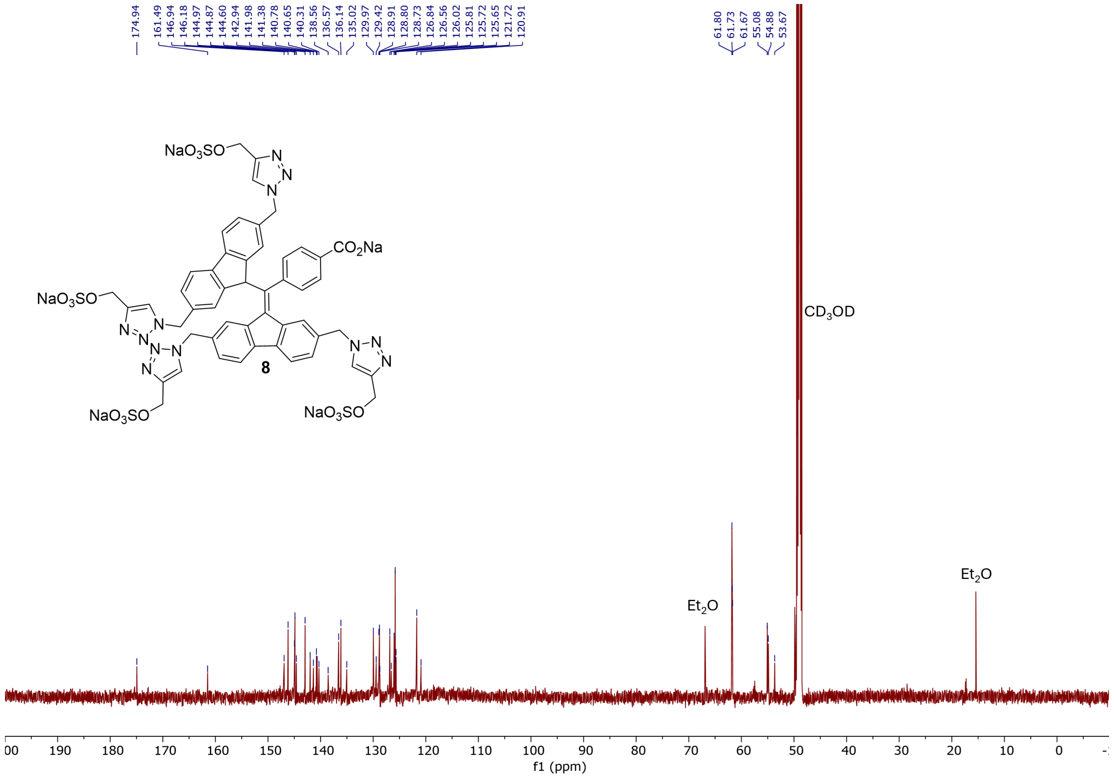

Figure S17. ^{13}C NMR spectrum of compound **8**.

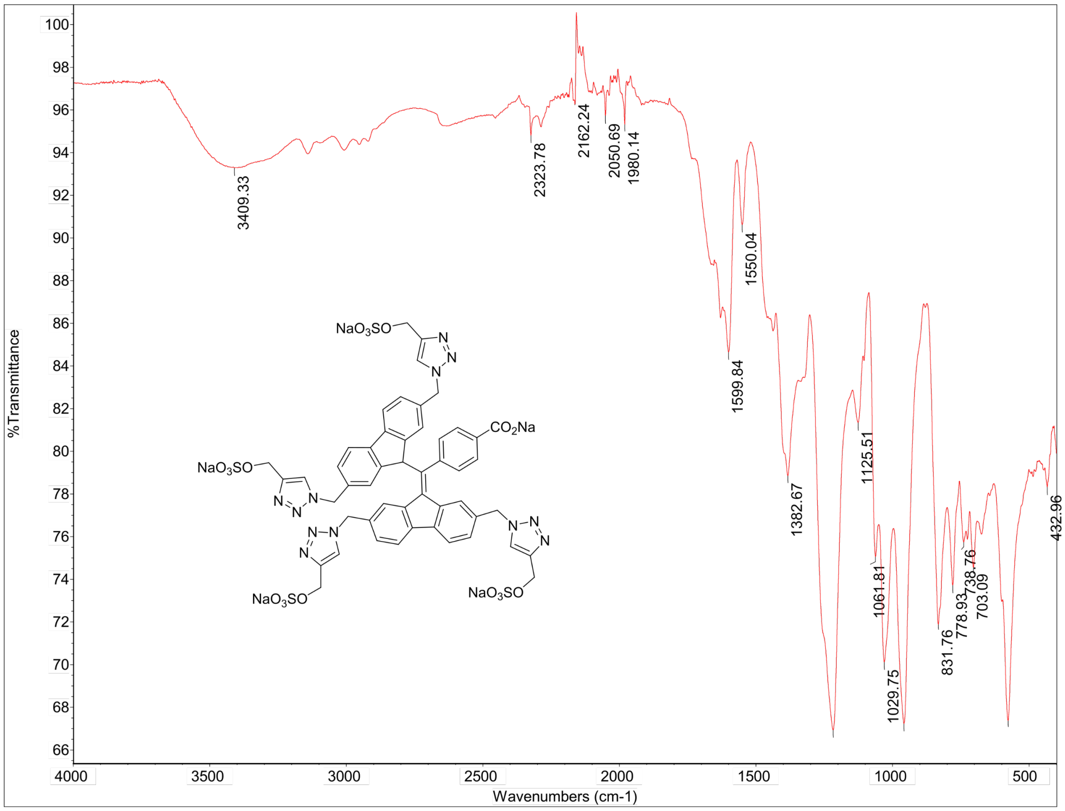

Figure S18. IR spectrum of compound **8**.

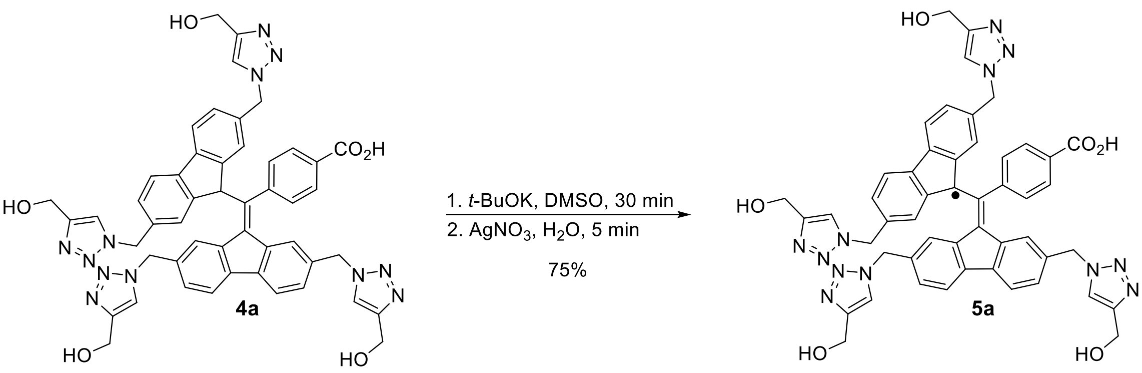

BDPA-OH radical 5a. To a solution of compound **4a** (20 mg, 0.022 mmol) in DMSO (1.0 mL), was added a freshly prepared solution of *t*-BuOK (0.176 mL, 0.044 mmol, 0.25M in DMSO) and the resulting solution was stirred at 23 °C for 30 min. A solution of $AgNO_3$ (18.8 mg, 0.110 mmol) in H_2O (0.1 mL) was added and the red solution was stirred for 5 min. The mixture was centrifuged, the solution was decanted and poured into Et_2O (10 mL), followed by centrifugation and decantation of the Et_2O layer. This was repeated until a precipitate was obtained and DMSO was removed. The precipitate was washed with H_2O (1 mL), centrifuged and decanted. The precipitate was dissolved in DMSO (0.5 mL), re-precipitated with Et_2O (10 mL) and dried to give **5a** (15 mg, 75%) as a reddish brown solid.

IR (ATR, cm^{-1}): $\tilde{\nu}$ 3308.25 (s,b) (O-H, alcohol), 3138.62 (O-H), 1581.72 (s) (C=O); HRMS (ESI): *m/z* calcd. for $C_{50}H_{41}N_{12}O_6$ [M-H^-] 904.3199, found 904.3186.

EPR (DMSO):

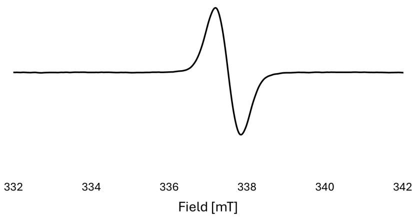

HPLC:

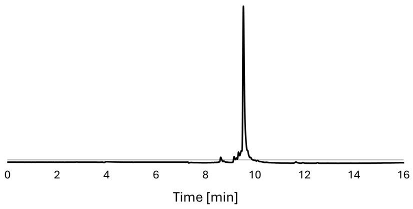

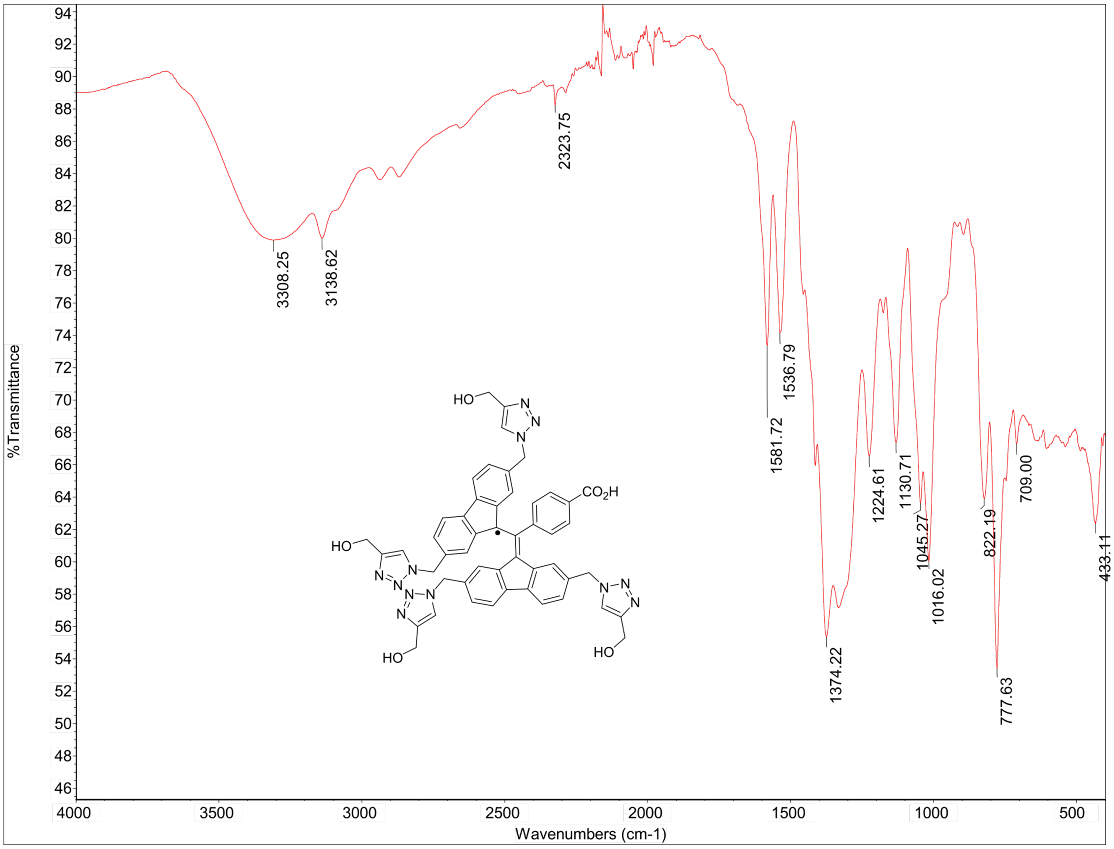

Figure S19. IR spectrum of compound **5a**.

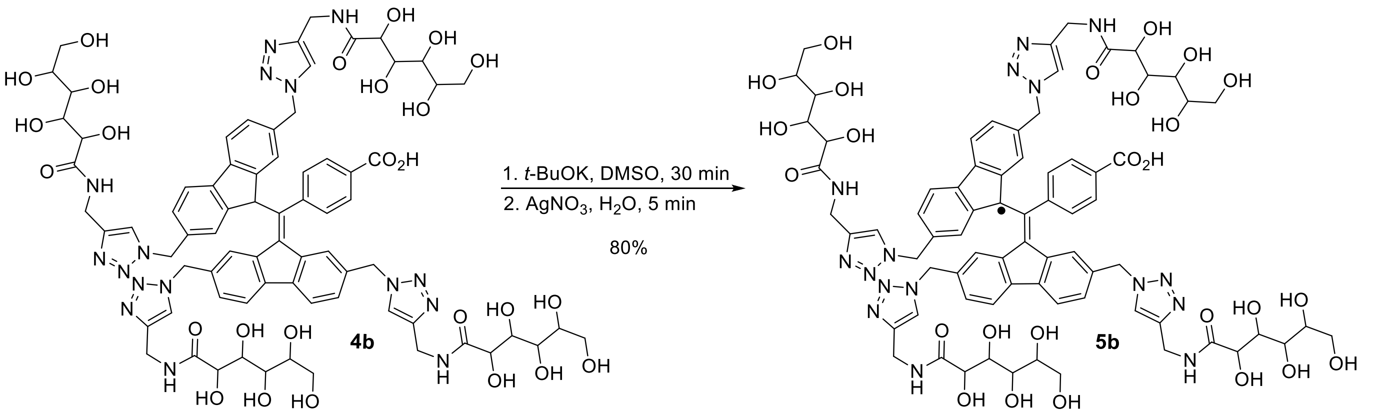

BDPA-hydroxyamide radical 5b. To a solution of compound **4b** (10 mg, 0.006 mmol) in DMSO (0.75 mL), was added a freshly prepared solution of *t*-BuOK (0.05 mL, 0.012 mmol, 0.25M in DMSO) and the resulting solution was stirred at 23 °C for 30 min. A solution of $AgNO_3$ (5.3 mg, 0.031 mmol) in H_2O (0.05 mL) was added and the red solution was stirred for 5 min. The mixture was centrifuged, the solution was decanted and poured into Et_2O (10 mL), followed by centrifugation and decantation of the Et_2O layer. This was repeated until a precipitate was obtained and DMSO was removed. The precipitate was washed with H_2O (1 mL), centrifuged and decanted. The precipitate was dissolved in DMSO (0.5 mL), re-precipitated with Et_2O (10 mL) and dried to give **5b** (8 mg, 80%) as a reddish brown solid.

IR (ATR, cm^{-1}): $\tilde{\nu}$ 3308.20 (s,b) (O-H, alcohol), 2932.20 (C-H), 1654.47 (s) (C=O), 1532.07 (s) (N-H), 1418.83 (m) (C-C, aromatic), 1072.40 (s) (C-O), 1022.53 (m) (C-N); HRMS (ESI): *m/z* calcd. for $C_{74}H_{86}N_{16}O_{26}$+$2Na^+$ [M+$2Na^+$] 830.2842, found 830.2676.

<u>EPR(DMSO):</u>

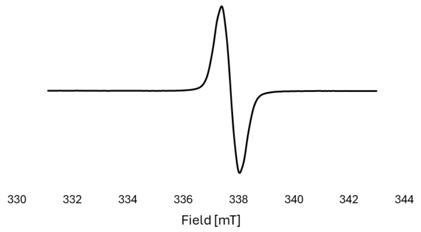

<u>HPLC:</u>

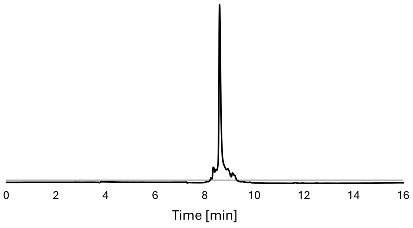

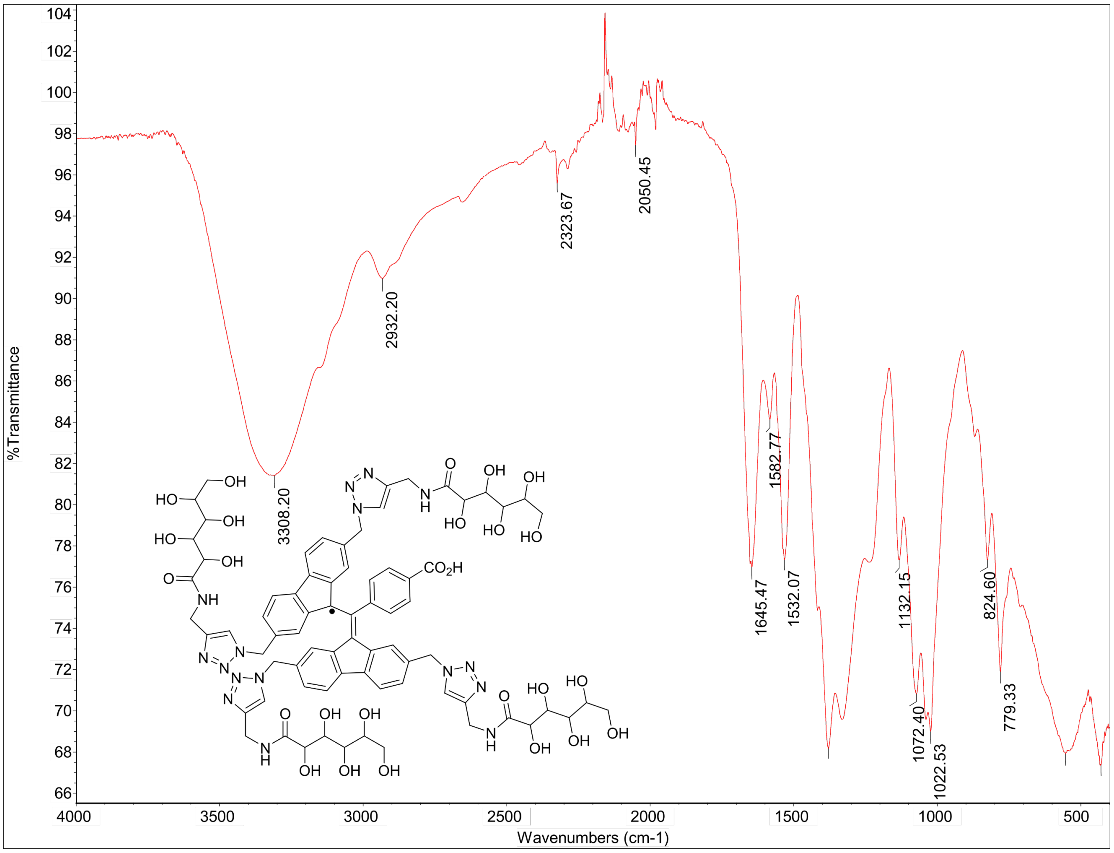

Figure S20. IR spectrum of compound **5b**.

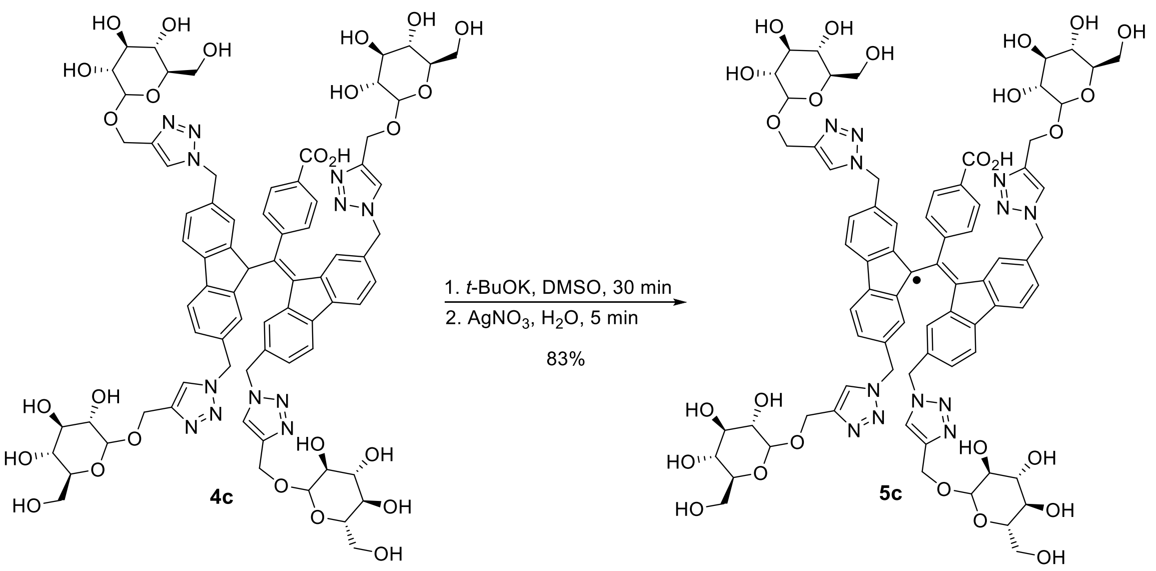

BDPA-glucose radical 5c. To a solution of compound **4c** (20 mg, 0.013 mmol) in DMSO (1.5 mL), was added a freshly prepared solution of *t*-BuOK (0.1 mL, 0.0257 mmol, 0.25M in DMSO) and the resulting solution was stirred at 23 °C for 30 min. A solution of $AgNO_3$ (10.9 mg, 0.0643 mmol) in H_2O (0.1 mL) was added and the red solution was stirred for 5 min. The mixture was centrifuged, the solution was decanted and poured into Et_2O (10 mL), followed by centrifugation and decantation of the Et_2O layer. This was repeated until a precipitate was obtained and DMSO was removed. The precipitate was dissolved in H_2O (0.5 mL), centrifuged and decanted. To the decanted solution, MeOH (2 mL) was added, followed by Et_2O (10 mL). The precipitate was collected by centrifugation, followed by decantation of the solvent and was dried to give **5c** (16.6 mg, 83%) as a reddish brown solid.

IR (ATR, cm^{-1}): $\tilde{\nu}$ 3353.00 (s,b) (O-H, alcohol), 2881.71 (C-H), 1583.61 (s) (C=O), 1374.82 (s) (C-N) 1038.58 (m) (C-O); HRMS (ESI): *m/z* calcd. for $C_{74}H_{81}N_{12}O_{26}$+2Na^+ [M+2Na^+] 799.7585, found 799.7495.

EPR (DMSO):

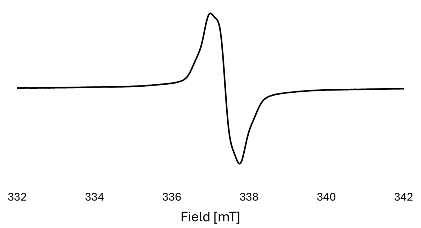

HPLC:

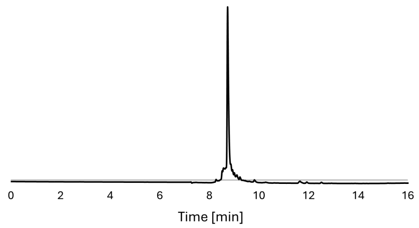

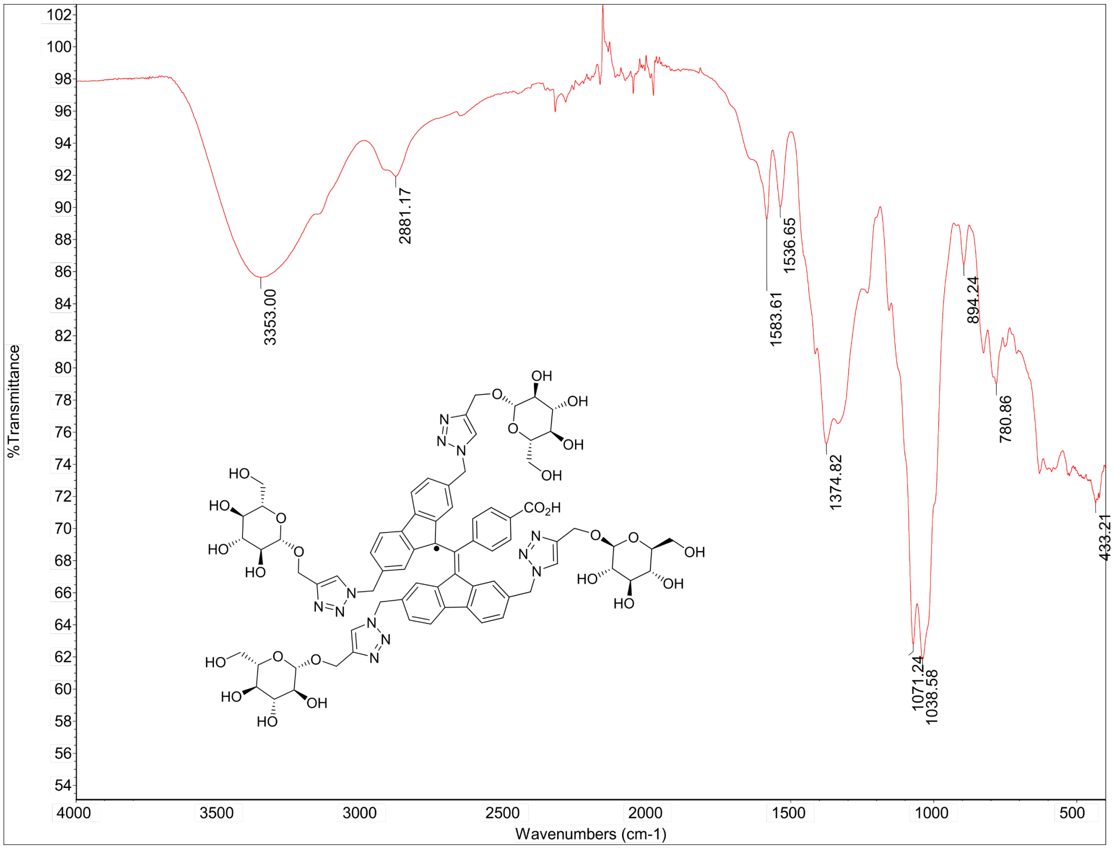

Figure S21. IR spectrum of compound **5c**.

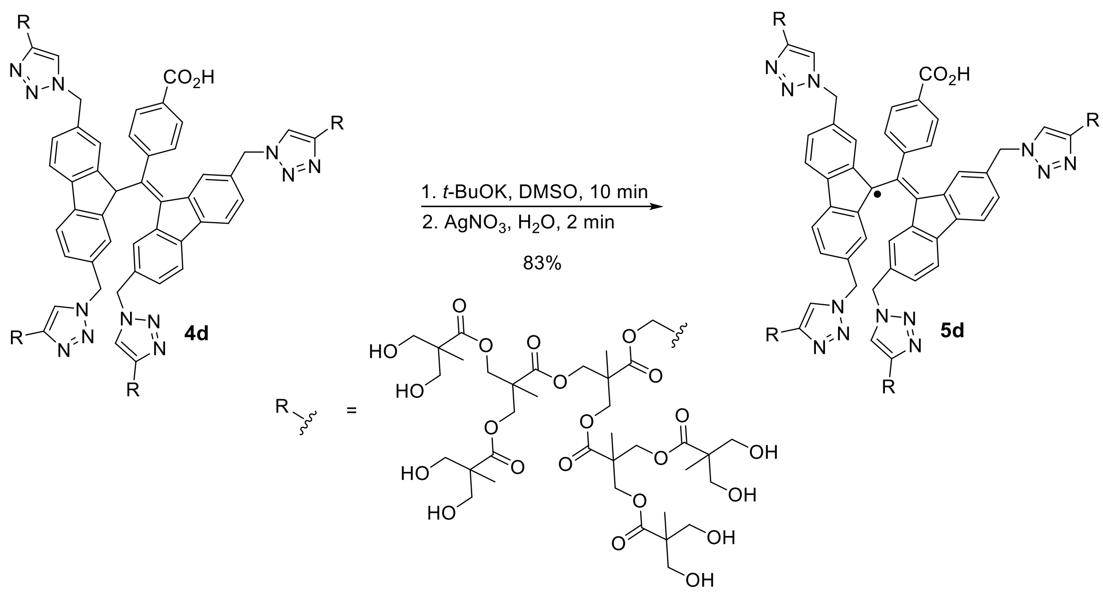

BDPA-dendrimer radical 5d. To a solution of compound **4d** (20 mg, 0.0048 mmol) in DMSO (1.5 mL), was added a freshly prepared solution of *t*-BuOK (0.04 mL, 0.0096 mmol, 0.25M in DMSO) and the resulting solution was stirred at 23 °C for 10 min. A solution of $AgNO_3$ (4 mg, 0.024 mmol) in H_2O (0.1 mL) was added and the red solution was stirred for 2 min. The mixture was centrifuged; the solution was decanted and poured into Et_2O (10 mL). The precipitate formed was collected by centrifugation followed by decantation of the solvent. The precipitate was redissolved in MeOH (1 mL) and re-precipitated by addition of Et_2O (10 mL) twice. The precipitate was dissolved in MeOH (1 mL) and centrifuged to remove the Ag residue and decanted and dried to give **5d** (14 mg, 70%) as a reddish brown solid.

IR (ATR, cm^{-1}): $\tilde{\nu}$ 3378.89 (s,b) (O-H, alcohol), 2882.93 (C-H), 1723.02 (s) (C=O, ester), 1461.22 (m) (C-C), 1031.54 (m) (C-O); ESI-HRMS: *m/z* calcd. for $C_{190}H_{265}N_{12}O_{90}$+4$Na^+$ [M+4Na^+] 1061.6524, found 1061.6410.

EPR (DMSO):

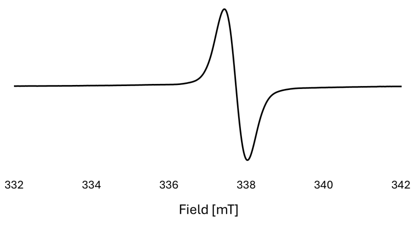

HPLC:

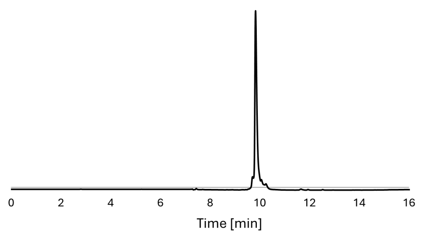

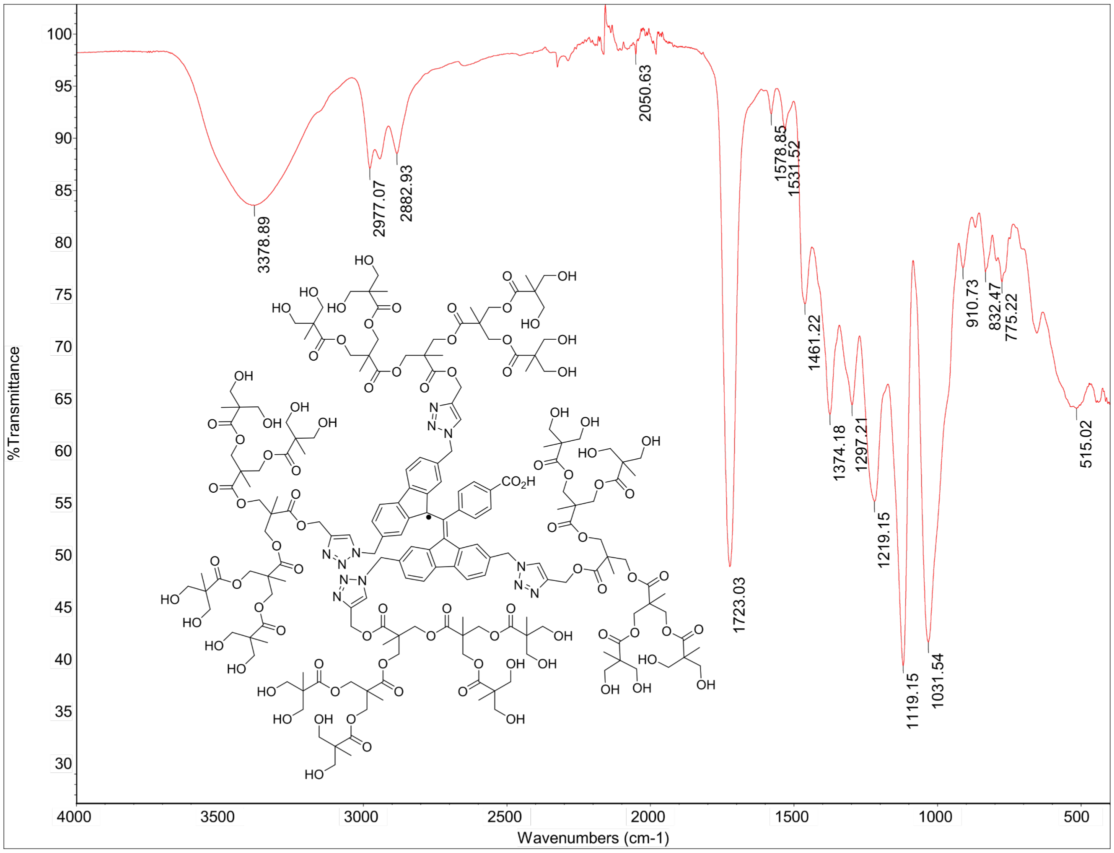

Figure S22. IR spectrum of compound **5d**.

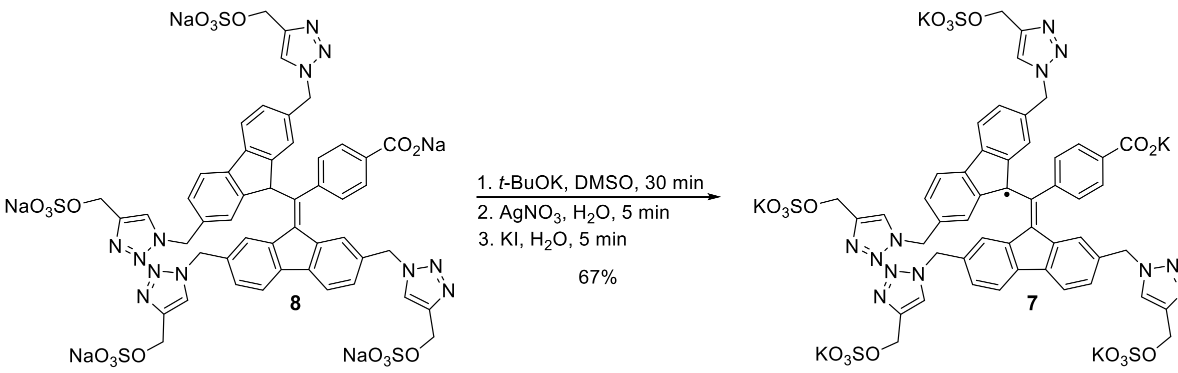

BDPA-sulfate radical 7. To a solution of compound **8** (20 mg, 0.015 mmol) in DMSO (1.0 mL), was added a freshly prepared solution of *t*-BuOK (0.12 mL, 0.029 mmol, 0.25M in DMSO) and the resulting solution was stirred at 23 °C for 45 min. A solution of $AgNO_3$ (12.7 mg, 0.075 mmol) in H_2O (0.06 mL) was added and the red solution was stirred for 5 min. A solution KI in H_2O (60 µL) was added, and the solution was stirred for 5 min. The mixture was centrifuged; the solution was decanted and poured into Et_2O (10 mL). The precipitate formed was collected by centrifugation followed by decantation of the solvent. The precipitate was redissolved in MeOH:H_2O (1:1) and re-precipitated by addition of Et_2O (10 mL) twice. The precipitate was dissolved in MeOH (1 mL) and centrifuged again to remove the Ag residue and decanted and dried to give **5d** (14 mg, 67%) as a reddish brown solid.

IR (ATR, cm^{-1}): $\tilde{\nu}$ 3442.06 (s,b) (O-H, alcohol), 2882.91 (C-H), 1593.14 (s) (C-C), 1382.67 (s) (S=O), 1216.95 (s) (C-O); ESI-HRMS: *m/z* calcd. for $C_{50}H_{37}N_{12}O_{18}S_4$ [M^{4-}] 305.2813, found 305.2828.

EPR (H_2O):

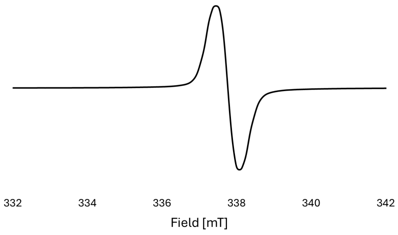

HPLC:

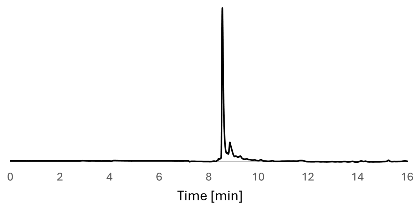

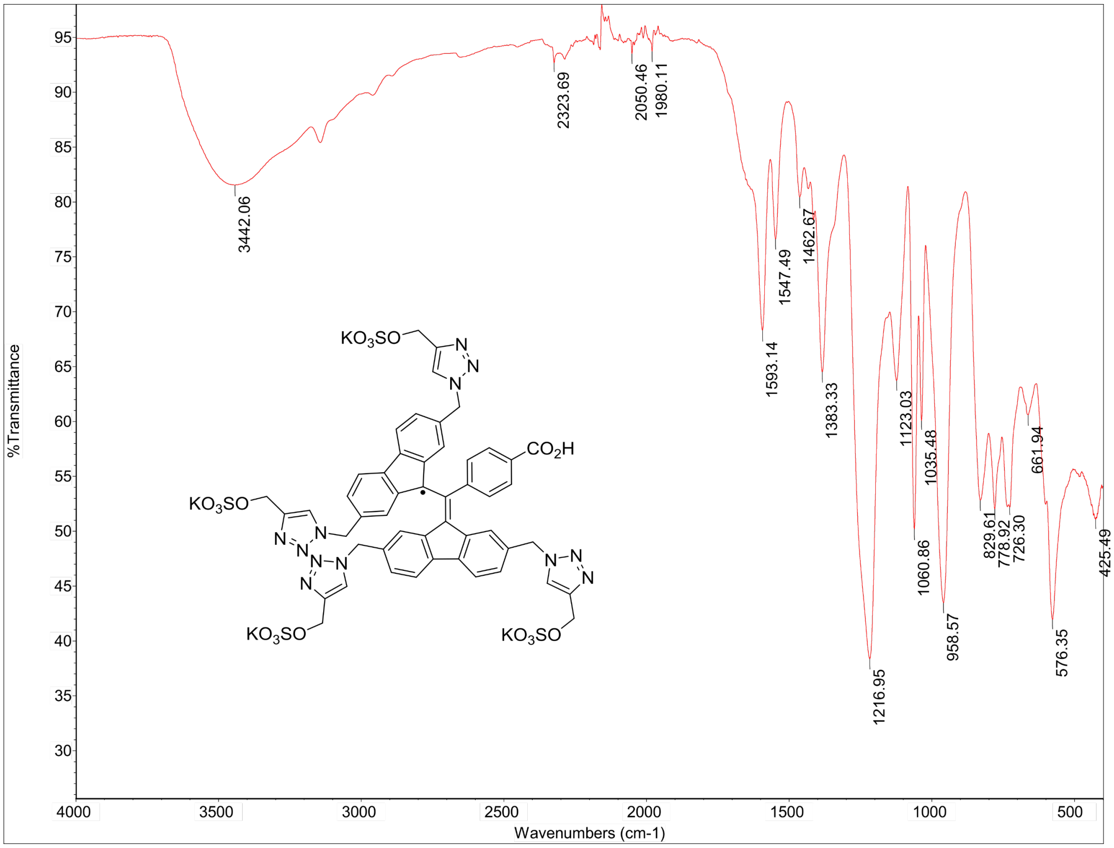

Figure S23. IR spectrum of compound **7**.

Quantification of BDPA radicals

The amount of radical present in the BDPA radicals **5a**-**d** and **7** was determined by spin counting using EPR spectroscopy. A stock solution of 4-hydroxy-TEMPO (1.0 M) was prepared in DMSO. The stock solution was diluted into a series of concentrations (0.1–1.5 mM), and each sample was measured by EPR spectroscopy. The area under the peaks of each spectrum, obtained by double integration, was plotted against its concentration to yield a standard curve. Solutions of BDPA radicals **5a**-**d** and **7** (1 mM) in DMSO were measured under same conditions and double-integrated to obtain the area under the peaks. Comparison with the TEMPO calibration curve showed that the radical content was approximately 83% (**5a**), 82% (**5b**), 80% (**5c**), 56% (**5d**) and 89% (**7**) with an error margin of 5–10%.

Persistence of BDPA-dendrimer 5d

Solvent-dependent persistence

The persistence of BDPA-dendrimer **5d** was investigated by monitoring its absorption at 503 nm using UV-vis spectroscopy. UV-Vis spectroscopy was used to monitor the radical persistence since it allowed easier and more accurate quantification of the radical than EPR spectroscopy. The concentrations of **5d** in H_2O, DMSO and glycerol were 10 mM (by weight) The solutions were kept at 23 °C and the UV-vis spectra were recorded at different time intervals by removing aliquots (3 µL) and diluting with H_2O (0.5 mL). Commercially purchased solvents were used without further drying and purification for these experiments, except for DMSO which was dried over molecular sieves (3 Å).

The UV-vis spectra of BDPA-dendrimer radical **5d** in DMSO, H_2O and glycerol as a function of time are shown in **Figure S24**. The characteristic radical absorption band between 440 – 550 nm (λ_{max} = 503 nm), decreases as the radical decomposes. In DMSO (**Figure S24A**), the absorbance of **5d** increases with time for about 3 days and then becomes constant. In H_2O, the absorbance decreases exponentially with time, yielding a half-life of about 2 days (**Figure S24B**). In glycerol, **5d** shows a gradual decrease in the absorbance at 503 nm, while the absorbance in the 360 - 440 nm region gradually increases with time, unlike the measurements in DMSO or H_2O. The normalized absorbance at 503 nm, as a function of time, shows a half-life of ca. 8 days in glycerol (**Figure S24C**).

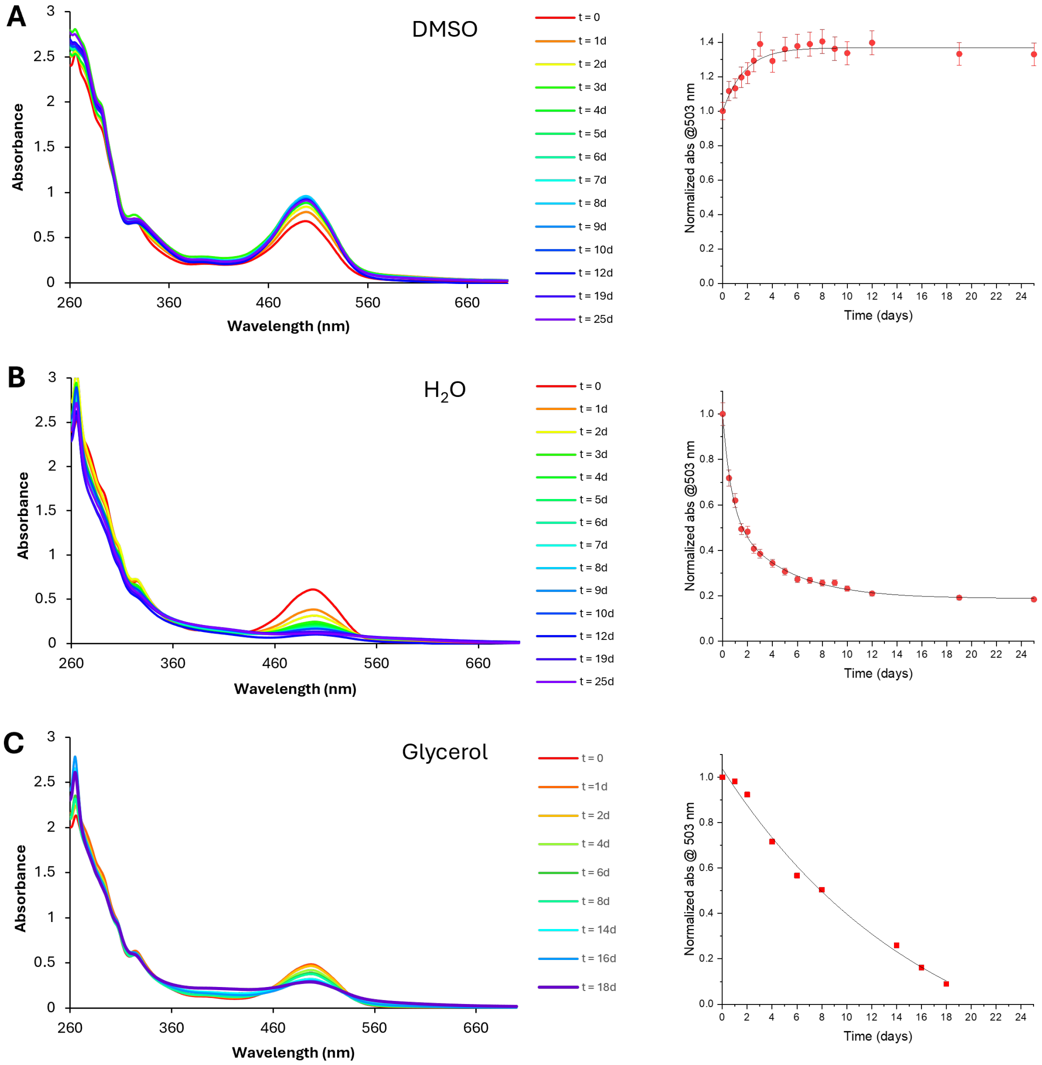

Figure S24. The UV-vis absorbance spectra of **5d** (left) and its corresponding normalized absorbance at 503 nm (right), plotted as a function of time in DMSO (**A**), H_2O (**B**) and glycerol (**C**).

One possible explanation of the unexpected increase of absorbance of **5d** in DMSO could be low solubility that might result in slow dissolution. To rule out this explanation, a 10 mM solution of **5d** in DMSO was prepared and an aliquot was immediately filtered through a 0.2 μm membrane filter. Its persistence was subsequently compared with the unfiltered solution (**Figure S25**). The material in the two solutions showed identical persistence curves, indicating that solubility of **5d** in DMSO was not responsible for the increase in absorbance of radical with time.

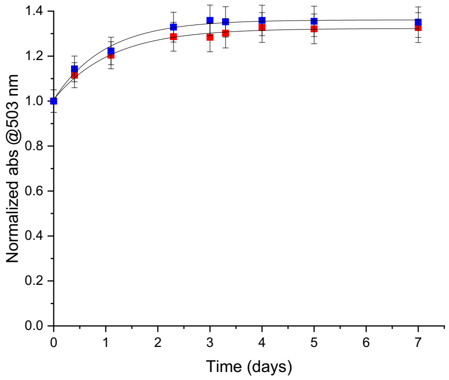

Figure S25. Comparison of persistence of filtered (■) and unfiltered (■) **5d** solution in DMSO (10 mM) at 23 °C, monitored by UV-vis spectroscopy at 503 nm.

We also determined whether the increase in absorbance was concentration dependent, by comparing the persistence of 5d at different concentrations (1 mM, 5mM, 10 mM and 20 mM, **Figure S26**). All concentrations exhibited similar persistence curve profiles, with each showing an initial increase in absorbance.

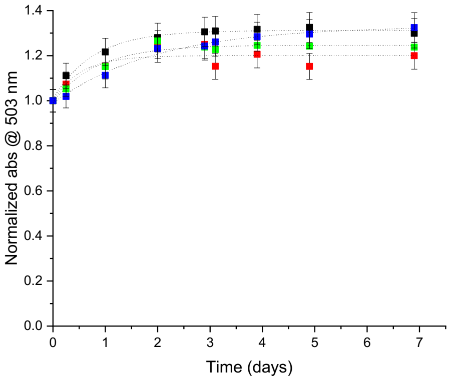

Figure S26. Persistence of 5d for 1 mM (■), 5mM (■), 10 mM (■) and 20 mM (■) solutions in DMSO at 23 °C monitored by UV-vis spectroscopy at 503 nm.

To determine if the initial increase in concentration of radical **5d** in DMSO originated from presence of the non-radical precursor **4d** in the sample of **5d**, a solution of **4d** in DMSO was monitored by UV-vis spectroscopy (**Figure S27A**). Initially, formation of the anion was observed (λ_{max} = 630 nm), which subsequently decreased as the radical gradually formed over a period of 4 days (**Figure S27A**,**B**). The increase in radical content for **5d** is shown for comparison (**Figure S27B**).

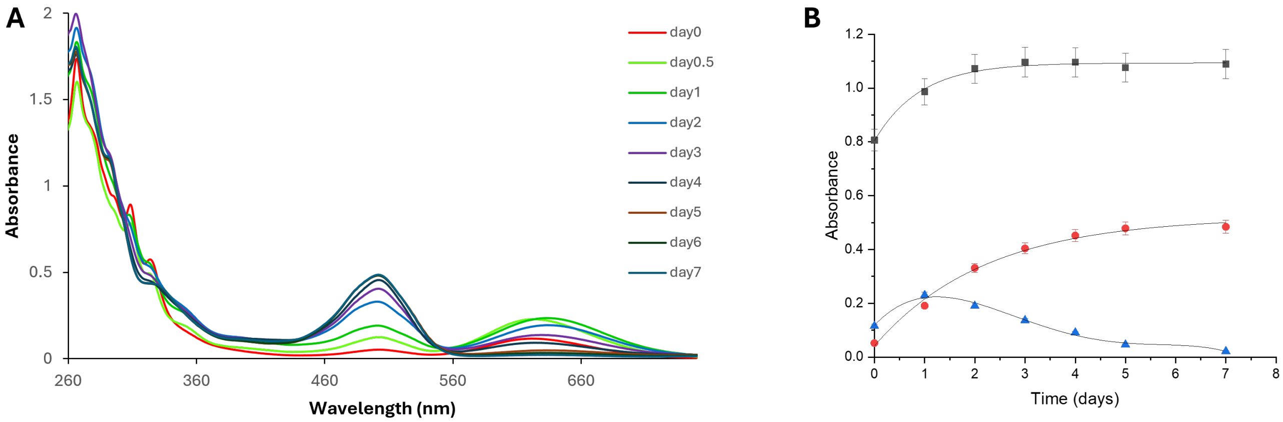

Figure S27. **A**. The UV-vis absorbance of BDPA-dendrimer **4d** in DMSO over 7 days at 23 °C. **B**. Comparison of effect of DMSO on radical **5d** (■) with its non-radical **4d**. Anion formed in non-radical is shown by (▲) while the radical formed over time in the **4d** is indicated by (●).

To assess whether dissolved oxygen in DMSO plays a role in the oxidation of the BDPA anion to the radical, conversion of **4d** in DMSO to the radical **5d** was monitored in the presence and absence of oxygen (**Figure S28**). The radical formation was comparable in both cases, indicating that dissolved oxygen is not required for radical formation.

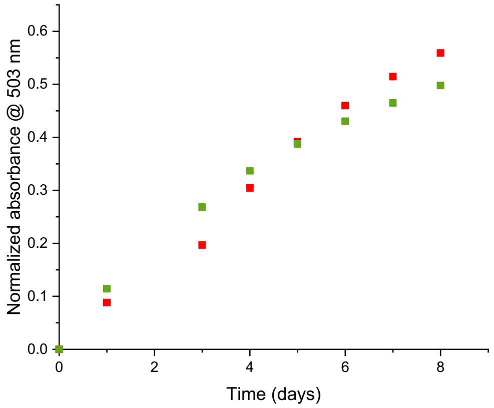

Figure S28. Normalized absorbance (503 nm) of **4d** in DMSO (10 mM) plotted as a function of time, in the presence (■) and absence of oxygen (■).

Liquid DNP NMR measurements

Experimental setup

^{1}H DNP NMR experiments were performed on a home-built DNP spectrometer, which was a modified Bruker Avance II wide-bore spectrometer operating at 9.4 T. Microwaves were generated by a 12 W gyrotron operating at 263 GHz.[4] The DNP probehead was a homemade Fabry-Perot/stripline double resonance structure operating at 263 GHz/400 MHz.[5] DNP-enhanced (mw on) and reference (mw off) NMR FID signals were recorded using standard 90° RF pulse excitation. The pulse length was 25 μs,

and the repetition time was optimized for each radical concentration and set to one second for each experiment. DNP enhancement was measured by integrating all glycerol protons. Enhancement was calculated according to $\varepsilon = (I/I_0) - 1$, where I and I_0 are the dynamic and Boltzmann nuclear polarizations, respectively. Temperature calibration was performed by monitoring the distance between the CH_2 and OH groups in the NMR spectrum, as previously described.[6]

J-band EPR experiments were carried out using an ELEXSYS E780 at temperatures of 298 K and 100 K with the following parameters for CW EPR: a microwave frequency of 262.75 GHz, a microwave power of 0.02377 mW, a sweep width of 20 mT, a field modulation frequency of 100 kHz, a field modulation amplitude of 0.3 mT, a time constant of 1 s, 201 points, and one scan. The echo detected (ED) spectrum was measured at 100 K using a pulse sequence of 70–250–140 ns with a repetition time of 100 ms. The number of shot per point was 10 ns, and the number of scans was 1.

The BDPA dendrimer radical **5d** concentration was between 10 and 40 mM, as determined by X-band CW EPR at room temperature **(Figure S28A)**. The J-band CW and ED EPR spectra are shown in **Figures S28B** and **S28C**, respectively.

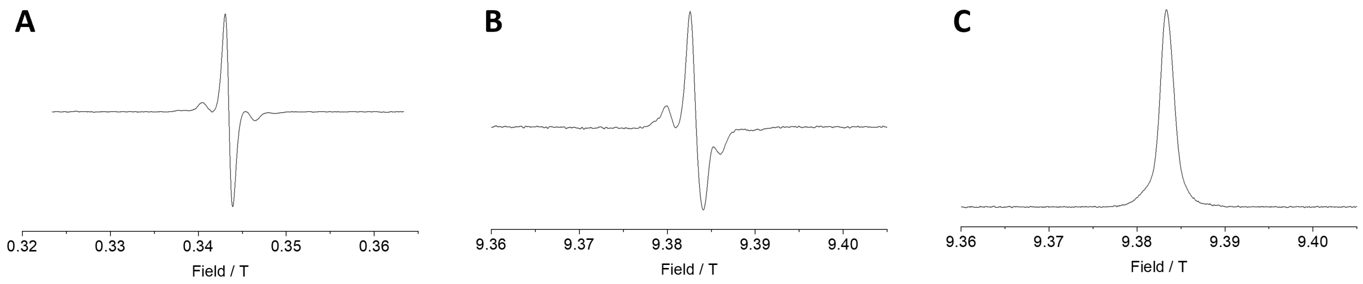

Figure S29. X-band and J-band EPR of BDPA-dendrimer. **A**. X-band CW EPR spectrum of 20 mM **5d** in glycerol at 298 K. **B**. J-band CW EPR of 20 mM **5d** in glycerol at 298 K. **C.** J-band ED EPR of 0.3 mM **5d** at 100 K.

^{1}H DNP Field Profile

The ^{1}H DNP field profile of the glycerol is antisymmetric (**Figure S29**), with peaks located at magnetic field strengths that are displaced by two nuclear Larmor frequency ($2\omega_H/\gamma_e$). This is characteristic of the SE mechanism.

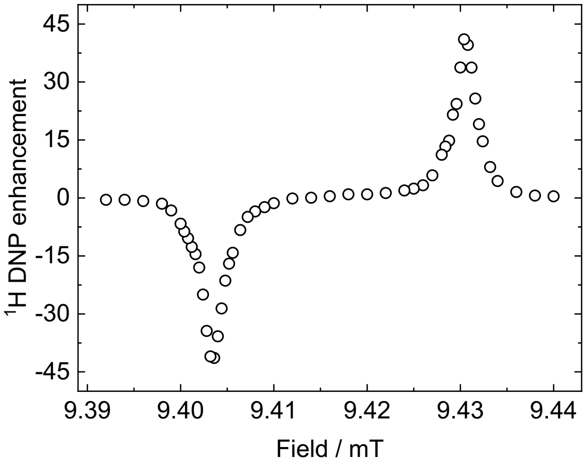

Figure S30. Field profile of the ^{1}H DNP enhancement for a solution of BDPA-dendrimer **5d** (20 mM) in glycerol at 9.4T and 315 K.

DNP measurements at different concentrations of 5d

To evaluate the effect of radical concentration on DNP performance, ^{1}H DNP was measured for **5d** at concentrations of 10, 20 and 40 mM, showing a clear concentration-dependent increase in DNP performance (**Table S1**).

Table S1. ^{1}H DNP enhancement at three different concentrations of **5d**.

Concentration	^{1}H DNP enhancement
10 mM	18 ± 3
20 mM	40 ± 5
40 mM	57 ± 6

References

1. S. Mandal and S. T. Sigurdsson, *Chem. Commun.*, 2020, **56**, 13121-13124.
2. E. H. Ryu and Y. Zhao, *Org. Lett.*, 2005, **7**, 1035-1037.
3. A. L. M. Morotti, K. L. Lang, I. Carvalho, E. P. Schenkel and L. S. C. Bernardes, *Tetrahedron Lett.*, 2015, **56**, 303-307.
4. V. Denysenkov, M. J. Prandolini, M. Gafurov, D. Sezer, B. Endeward and T. F. Prisner, *Phys. Chem. Chem. Phys.*, 2010, **12**, 5786-5790.
5. V. Denysenkov, D. H. Dai and T. F. Prisner, *J. Mag. Res.*, 2022, **337**.
6. A. A. Kuzhelev, V. Denysenkov, I. M. Ahmad, O. Y. Rogozhnikova, D. V. Trukhin, E. G. Bagryanskaya, V. M. Tormyshev, S. T. Sigurdsson and T. F. Prisner, *J. Am. Chem. Soc.*, 2023, **145**, 10268-10274.
